# High Throughput Transcriptome Profiling of Lithium Stimulated Human Mesenchymal Stem Cells Reveals Priming towards Osteoblastic Lineage

**DOI:** 10.1371/journal.pone.0055769

**Published:** 2013-01-30

**Authors:** Neeraj Kumar Satija, Deepa Sharma, Farhat Afrin, Rajendra P. Tripathi, Gurudutta Gangenahalli

**Affiliations:** 1 Stem Cell & Gene Therapy Research Group, Institute of Nuclear Medicine & Allied Sciences, Brig. S K Mazumdar Marg, Timarpur, Delhi, India; 2 Department of Biotechnology, Hamdard University, Hamdard Nagar, New Delhi, India; Univ.Mass.Med School, United States of America

## Abstract

Human mesenchymal stem cells (hMSCs) present in the bone marrow are the precursors of osteoblasts, chondrocytes and adipocytes, and hold tremendous potential for osteoregenerative therapy. However, achieving directed differentiation into osteoblasts has been a major concern. The use of lithium for enhancing osteogenic differentiation has been documented in animal models but its effect in humans is not clear. We, therefore, performed high throughput transcriptome analysis of lithium-treated hMSCs to identify altered gene expression and its relevance to osteogenic differentiation. Our results show suppression of proliferation and enhancement of alkaline phosphatase (ALP) activity upon lithium treatment of hMSCs under non-osteogenic conditions. Microarray profiling of lithium-stimulated hMSC revealed decreased expression of adipogenic genes (CEBPA, CMKLR1, HSD11B1) and genes involved in lipid biosynthesis. Interestingly, osteoclastogenic factors and immune responsive genes (IL7, IL8, CXCL1, CXCL12, CCL20) were also downregulated. Negative transcriptional regulators of the osteogenic program (TWIST1 and PBX1) were suppressed while genes involved in mineralization like CLEC3B and ATF4 were induced. Gene ontology analysis revealed enrichment of upregulated genes related to mesenchymal cell differentiation and signal transduction. Lithium priming led to enhanced collagen 1 synthesis and osteogenic induction of lithium pretreated MSCs resulted in enhanced expression of Runx2, ALP and bone sialoprotein. However, siRNA-mediated knockdown of RRAD, CLEC3B and ATF4 attenuated lithium-induced osteogenic priming, identifying a role for RRAD, a member of small GTP binding protein family, in osteoblast differentiation. In conclusion, our data highlight the transcriptome reprogramming potential of lithium resulting in higher propensity of lithium “primed” MSCs for osteoblastic differentiation.

## Introduction

Human mesenchymal stem cells are an attractive target for cell-based therapies due to their ease of isolation, in vitro expansion, differentiation potential and immunomodulatory effects [1]. Present in the bone marrow, they give rise to osteoblasts and have been exploited for treating orthopedic defects and disorders such as long bone defects [2] and osteoporosis [3] owing to slow or inability of natural repair mechanisms. Hence, approaches like co-transplantation with factors like BMPs and genetic modification [4] are being evaluated to accelerate bone healing by stimulating both transplanted as well as endogenous stem cells. This suggests the need for the development of novel, simpler and inexpensive strategies to promote osteogenesis to meet the growing requirement of orthopedic patients.

The canonical Wnt signaling is demonstrated to play a major role in determining the fate of MSCs favouring their differentiation into osteoblasts [5]. Glycogen synthase kinase-3β (GSK-3β) acts as a negative regulator of Wnt signaling by phosphorylating β-catenin resulting in its degradation by the ubiquitin-proteasome system [6]. Lithium, which has been in clinical use for years for the treatment of psychiatric disorders, is a potent inhibitor of GSK-3β and is able to mimic Wnt signaling [7]. Studies in mice models exhibiting low bone mass, osteoporosis [8] and cleidocranial dysplasia [9] have demonstrated enhanced osteogenesis upon lithium administration.

Few studies have, however, evaluated the effect of lithium use on bone among patients on lithium therapy [10]–[12], but reported contradictory results. We therefore undertook microarray profiling of hMSCs stimulated with lithium for short time period (7 days) to decipher the changes induced in the transcriptome and provide a molecular basis for lithium’s action in regulating osteogenic fate of hMSCs. Lithium chloride was found to reduce the proliferation rate and upregulated alkaline phosphatase (ALP) activity while suppressing adipogenic, osteoclastogenic and immune response genes. The transcriptome reprogramming by lithium affected osteogenic genes and osteogenic induction of lithium ‘primed’ cells was enhanced. However, RNAi-mediated silencing of RRAD significantly reduced lithium’s priming potential.

## Materials and Methods

### MSC isolation & culture

Bone marrow aspirates (2–3 ml) of normal healthy donors were kindly provided by Brig. Velu Nair, Department of Hematology and Bone Marrow Transplantation, Army Research & Referral Hospital, New Delhi. Verbal consent was obtained from donors who volunteered since the cells were used only for *in-vitro* lab work. The committee approved the method, however, as per the committee’s recommendation the details of the donors such as identity, age, sex, disease state and HIV status have been documented and maintained for records. This study was approved by the Institutional Committee on Stem Cell Research and Therapy of Institute of Nuclear Medicine and Allied Sciences. Mononuclear cells isolated from BM aspirates using Histopaque density gradient were plated at 0.1–0.5×10^6^ cells/cm^2^ in α-MEM (Sigma) containing 16.5% FBS (Gibco), 1% Streptomycin/Penicillin/amphoterecin (SLI) and 2 mM L-Glutamine (expansion/growth medium) [13]. Medium was changed after 48 h to remove non-adherent cells and thereafter every 3–4 days. MSCs were expanded at low plating density (50–500 cells/cm^2^) and cryopreserved. For experiments, early passage cells (passage 2–5) were used at the indicated densities.

### MSC characterization

Cells were characterized by flow cytometry for surface antigens: CD44, CD105, VCAM-1 (Santa Cruz Biotechnology) and CD34 (Calbiochem).

To assess the differentiation potential, MSCs were plated (1000 cells/cm^2^) in 24 well plate and grown to confluence. For adipogenic differentiation, confluent monolayer was cultured in adipogenic medium (expansion medium supplemented with 0.5 µM dexamethasone, 60 µM indomethacin and 0.5 mM IBMX) [14] for 15 days, and lipid-laden adipocytes were observed microscopically upon staining with Oil Red-O stain. For osteogenic differentiation, osteogenic medium (expansion medium supplemented with 10^−9^ M dexamethasone, 10 mM β-glycerophosphate and 0.2 mM ascorbic acid) was added to confluent well. After 21 days, staining for mineralization was performed by von Kossa and Alizarin Red S methods.

### CFU-F assay

MSCs were plated at very low density, 100 cells per 60 mm dish (in triplicate) and cultured for 2 weeks. LiCl at respective concentrations was added only once, the day following plating the cells. After 14–15 days, dishes were washed with PBS and stained with 0.5% (w/v) crystal violet and colonies consisting of more than 50 cells were counted.

### Proliferation assay

Cells were plated at density of 500 cells/cm^2^ and stimulated with various concentrations of LiCl. Cell proliferation was assessed using MTT on day 6. Briefly, MTT was added to wells at final concentration of 0.5 mg/ml and incubated for 4 h. After incubation, medium was discarded, wells washed with PBS and formazan crystals dissolved in DMSO. The plate was read at 450 nm and 630 nm in microplate reader (BioTek).

### ALP staining and activity assay

For ALP staining assay, cells were plated in 12 well plate (20000 cells/well) and stimulated with lithium (0, 5, 20 mM). Medium was changed every alternate day and cells stained for ALP using BCIP/NBT on 7^th^ day. For ALP activity assay, cells were plated at density of 5000 cells/cm^2^ (since high density is required for differentiation) in 96 well plate in quadruplet. Cells were stimulated with various concentrations (0–20 mM) of LiCl under growth conditions. Medium containing respective concentrations of LiCl was changed every alternate day for 7 days. On 7^th^ day, cells were washed in TBS and lysed in 0.1% Triton X-100 in TBS. An aliquot of the lysate was used for ALP activity assessment using para-nitrophenol phosphate (in 2-amino-2-methyl-1-propanol buffer, pH 10.5) as substrate, and the protein content was determined by Bradford method. ALP activity was normalized to protein content and expressed as pmol/min/mg of protein.

### Immunofluorescence staining

Cells were seeded on glass coverslips in 12 well plate and stimulated with 5 mM LiCl. Following stimulation, cells were washed with PBS, fixed in 4% paraformaldehyde at RT for 15 min. Cell were permeabilised and blocked in 0.1% Triton X-100, 1% BSA in PBS for 30 min at RT. Following washes with PBS, rabbit anti-β-catenin antiserum (1∶1000; Sigma) or anti-collagen I (1∶100; Santa Cruz Biotechnology) was added and incubated overnight at 4°C. After 3 washes with PBS, cells were incubated with FITC-conjugated secondary antibody for 1 h at RT, and washed 3 times with PBS for 5 min each. Cells were counterstained with nuclear stain DAPI for subcellular distribution study, and observed under confocal microscope (Olympus FV1000). Images for collagen staining were acquired under fluorescence microscope (Olympus IX51) using ImagePro keeping the image acquisition time constant.

### siRNA transfection

Cells were seeded at density of 5000/cm^2^ and transfected the following day. Transfection was done using Lipofectamine 2000CD (Invitrogen) and the mix was prepared in siRNA transfection medium (Santa Cruz Biotechnology). siRNA targeting RRAD and CLEC3B were purchased from Santa Cruz Biotechnology, ATF siRNA [15] was synthesized from Eurofins MWG and Mission Universal negative control siRNA 1 was procured from Sigma. CLEC3B siRNA was used at final concentration of 40 nM while others were used at 80 nM. Six hours post transfection, lithium was added to cells to a final concentration of 5 mM with medium change every alternate day till 7^th^ day. A second round of transfection was again performed 4 days after 1^st^ transfection. Cells were used on 7^th^ day for ALP activity assessment, however, for gene expression study cells were switched to osteogenic medium on 7^th^ day from 1^st^ transfection for 3 days.

### cDNA synthesis, PCR and Quantitative PCR

RNA was isolated using GenElute Mammalian Total RNA kit (Sigma) and cDNA synthesis was performed using RevertAid First strand cDNA synthesis kit (Fermentas) as per manufacturer’s instructions. Briefly, 100–500 ng RNA was reverse transcribed using MMuLV reverse transcriptase and random hexamers at 42°C for 60 min. 1µl of cDNA was used per 25 µl reaction using Maxima SYBR Green real time PCR master mix (Fermentas) and gene specific primers (Table S1). qPCR cycling conditions were preincubation at 95°C for 10 min, followed by 40 amplification cycles (95°C for 15 sec, 56–62°C (depending on primer) for 30 sec, 72°C for 30 sec) on ABI 7500 (Applied Biosystems) or Mx3005 (Stratagene) or Cfx96 (Bio-Rad). This was followed by dissociation curve analysis. Data was analysed by comparative ΔΔC_t_ method using 18S rRNA as an internal control. All reactions were setup in duplicate. For PCR, 1 µl cDNA was used with gene specific primers (Table S2). PCR cycling conditions were preincubation at 94°C for 2 min, followed by amplification cycles (94°C for 30 sec, 55–65°C (depending on primer) for 30 sec, 72°C for 30 sec) on thermal cycler (MJ Research). Image processing was performed using ImageJ (NIH) and Adobe photoshop.

### Microarray

hMSC from three donors were plated in T75 flask or 100 mm petridishes (BD Falcon) at a density of 5000 cells/cm^2^. LiCl (5 mM) stimulation was continued for 7 days with medium change every alternate day. Cells were harvested by trypsinisation and stored in RNAlater (Ambion) to be shipped for microarray analysis. RNA isolation, quality control and hybridization were performed by Genotypic Technologies Pvt Ltd, Bangalore. Briefly, RNA was isolated using RNeasy Mini Kit (Qiagen) and quantified in Nanodrop Spectrophotometer. RNA sample purity ratios were more than 1.9 for 260/280 nm and 260/230 nm, and RIN (RNA integrity number) values were greater than 8.5 as evaluated on Bioanalyzer 2100 (Agilent Technologies). Following reverse transcription of RNA into cDNA, Cy3-labeled cRNA was produced by in vitro transcription and hybridized to human whole genome array chip (Human Whole Gene Expression Microarray, 4×44 K array, Agilent Technologies). Images were scanned and signal data acquired using feature extraction software (v10.5, Agilent technologies). The data is deposited in GEO database (accession no.: GSE34747).

### Microarray Expression Data analysis

Transcript probes which were reliably detected in all the replicates were considered for subsequent data analysis. The data was normalized by percentile shift normalization method using GeneSpring GX11 (Agilent technologies). Following normalization, average signal intensity of the probes showing atleast 30% change in expression across the 3 donors were computed and ratios (treatment/control) were log_2_ transformed. Statistical analysis of the data was performed using Cyber-T regularized t-statistic [16] due to small sample size (n = 3) since it takes into account Bayesian estimate of variance by pooling across genes with similar intensities [17]. Differentially regulated genes were identified based on average log_2_ ratio ≥±0.6 (±1.5 fold) and p value ≤0.05.

To identify biological processes and molecular functions represented by the differentially regulated genes, Functional annotation tool of the DAVID Bioinformatics Resources 6.7 was used [18] considering a p-value ≤0.05 as significant. Further, the signaling pathways regulated by significantly altered genes were identified using the tool Pathway Miner [19], which provides annotation from KEGG, Biocarta and GenMAPP, taking Fisher exact p-value≤0.05 as significant due to the small number of replicates [20].

### Western blotting

Whole cell lysates were prepared with RIPA buffer, incubated for 30 min on ice and centrifuged at 15000 g for 20 min. Protein content was determined by BCA method (Thermo Scientific). Protein (30 µg) was resolved on 12% SDS-PAGE and transferred onto nitrocellulose membrane (Pall Life Science). The membrane was blocked in 3% BSA in TBS buffer containing 0.1% tween-20 (TBST) for 1 h at RT and incubated overnight at 4°C with primary antibodies: pGSK3β Ser9 (1∶1000; Cell Signaling Technologies) and α-tubulin (50 ng/ml; Calbiochem). Following atleast three 5 min washes with TBST, membranes were incubated with HRP-conjugated secondary antibodies (1∶2000) for 1 h at RT and washed 3 times with TBST. Blots were developed using TMB stabilized substrate (Promega).

### Statistical analysis

Results are expressed as mean ± SD. For assays, statistical significance of differences between means was assessed using Student’s t-test considering p value≤0.05 as significant. For microarray data analysis, regularized t-statistic was used for identification of differentially regulated genes and Fisher exact t-test for gene ontology and pathway analysis.

## Results

### MSC culture and characterization

MSC cultures were established from BM aspirates of three donors by expanding cells at low density to enrich for early progenitors and maximally retain their multipotentiality [14], and achieve higher fold expansion [21] enabling use of early passage cells [22] for experiments. These cells expressed high levels of CD44 and CD105, low level of VCAM-1 (CD106) and did not express the hematopoietic marker CD34 (Figure S1A). Functional validation using osteogenic and adipogenic supplements demonstrated their differentiation into osteoblast and adipocytes (Figure S1B), thus ascertaining MSC character of the established cells.

### Inhibition of hMSC proliferation by lithium

**Figure 1:**
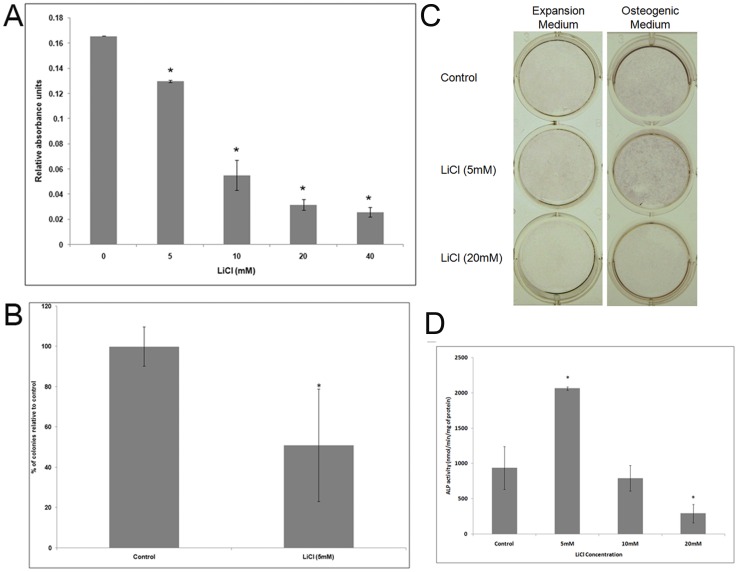
Effect of lithium on hMSC proliferation and osteoblast differentiation. (**A**) Cell number assessed by MTT assay and expressed as relative absorbance units. Student t-test applied using respective day controls. (**B**) CFU-F assay. Percentage of colonies formed relative to control is plotted. Data presented as mean±SD. (**C**) ALP staining of hMSCs under expansion and osteogenic conditions at different concentrations of lithium. (**D**) ALP activity. Data from one MSC sample representative of other donors is presented as mean±SD. *p≤0.05.

Investigation of the effect of lithium on hMSC revealed significant reduction of cell proliferation (Figure 1A). LiCl at 5 mM resulted in more than 20% reduction while at 40 mM it had maximum inhibitory effect with more than 80% reduction in cell proliferation.

CFU-F assay was also performed to reconfirm lithium’s effect since reduction of MTT to formazan depends on metabolic activity, particularly glycolysis [23] which is likely to be affected by GSK-3β inhibition, and might not give a true measure of cell numbers. LiCl at 5 mM significantly reduced number of colonies by approximately 50% (Figure 1B), while higher concentration (10 mM and above) completely inhibited colony formation.

### Increase in ALP activity on lithium stimulation

As ALP is a well established early osteogenic marker, lithium stimulation of hMSC for 7 days in the presence of expansion and osteogenic medium demonstrated highly inhibitory effect of 20 mM LiCl as no ALP staining was observed (Figure 1C). However, lower concentration (5 mM) resulted in enhanced ALP staining under both culture conditions. Quantification of ALP activity under expansion condition revealed consistent and significant enhancement in ALP activity in hMSCs from the donors at 5 mM lithium concentration (Figure 1D) and was, henceforth, selected for subsequent studies.

### LiCl stimulates nuclear translocation of β-catenin and inhibits GSK-3β

**Figure 2:**
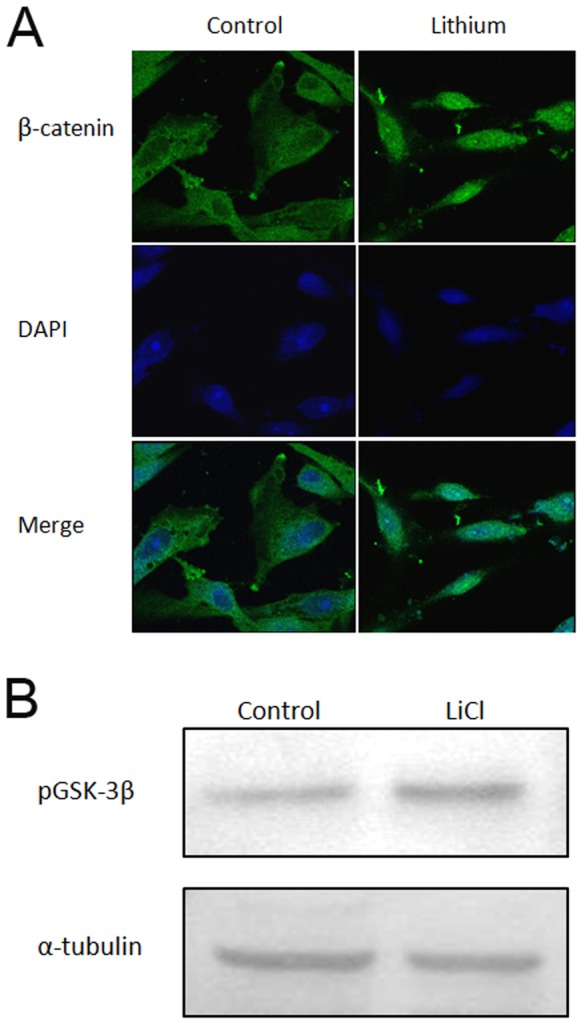
Wnt signaling induction on lithium treatment of hMSCs. (**A**) Subcellular distribution of β-catenin. β-catenin staining in green (FITC) and nuclei stained with DAPI (Blue). (**B**) Inhibitory phosphorylation of GSK-3β. α-tubulin used as loading control.

Since LiCl is known to inhibit GSK-3β [24] and activate Wnt signaling, we monitored the intracellular distribution of β-catenin whose nuclear translocation is indicative of active Wnt signaling [25]. hMSC stimulation with LiCl (5 mM) for 24 h in expansion medium resulted in enhanced nuclear staining for β-catenin (Figure 2A) suggesting activation of Wnt signaling. Further, pGSK-3β Ser9 levels were increased following 24 h lithium (5 mM) treatment of hMSCs compared to control (Figure 2B).

### Transcriptional response to lithium

The ability of LiCl to enhance ALP activity prompted us to undertake gene expression profiling of lithium stimulated hMSCs to elucidate the changes in the transcriptome likely favouring osteogenic differentiation. Due to the inherent genetic variability among the donors which can account for changes in gene expression, we elected to analyse the RNA independently from each donor rather than pooling them together in order to identify genes most likely affected by lithium treatment, which would emerge above the biological variability [26]. Though there were differences in the degree of response of a particular gene among the donors, we observed the correlation coefficient of intensity among all the 3 donors to be in range of 0.96–0.98 under our experimental conditions.

Using the selection criteria (log_2_ fold change ≥±0.6, p≤0.05), we found 93 genes were upregulated and 310 genes were downregulated on lithium treatment (Table S3). Among the differentially regulated genes, PLA2G4A (-1.4 fold) [27], CRIP1 (+5.39 fold) [28], ATF4 (+1.78 fold) and RRAD (+9.19 fold) [29] have been previously identified to be modulated in response to lithium in different cell types. Genes involved in cell adhesion (LAMA4, ITGA2, CLDN20), transport (TRPV6, GRIA3, SLC38A2, SLC22A3, CACNG6), transcription (EGR3, TSC22D1, BSX, HNF1A, FOXP1, RXRG, ETV1), metabolism (HK3, GK, AMY2B, ALDOB, KYNU, UPP1, PDE4D, PLD1, SULT1A2, PTGES), apoptosis (FAIM2, CARD17), tissue development (FGF7, NRG1, CHAT, TFPI2, ERRFI1, CYP26C1) and cellular homeostasis (BDKRB2, TXNDC2, NHLRC2) were altered upon lithium exposure.

#### Alteration of mesenchymal stem cell differentiation genes

Since lithium resulted in an increase in ALP activity, we sought to determine which of the differentially regulated genes play role in MSC differentiation. LiCl treatment of hMSCs suppressed FOXP1, ETV1, HTR7, ITGA2, VEGF and HGF which undergo downregulation on induction of differentiation [30] as well as stemness genes [31] undifferentiated embryonic cell transcription factor 1 (UTF1) (Table S3) [32] and nanog (-1.4 fold, p = 0.14). Adipogenesis promoting chemerin receptor, CMKLR1, and transcription factor CEBPA were downregulated whereas chondrogenic inhibitor GAS6 (growth arrest-specific 6) [33] was increased.

Expression of a number of transcription factors involved in osteogenic differentiation was modulated by LiCl under non-osteogenic conditions. For instance, ATF4 playing role in mineralization was upregulated while transcription factors TWIST1, PBX1 (-1.4 fold, p = 0.078) and TBX3 (-1.32 fold, p = 0.097) which negatively regulate osteoblast differentiation were downregulated. Other genes implicated in bone and skeletal development such as extracellular matrix protein tetranectin (CLEC3B, +3.89 fold, p = 0.088), BMP4 (+1.6 fold, p = 0.284) and endothelin1 were upregulated, whereas WWTR1, COL12A1, PTGS2 and stanniocalcin-1 (STC1) were observed to be downregulated.

### Validation of microarray data

**Figure 3:**
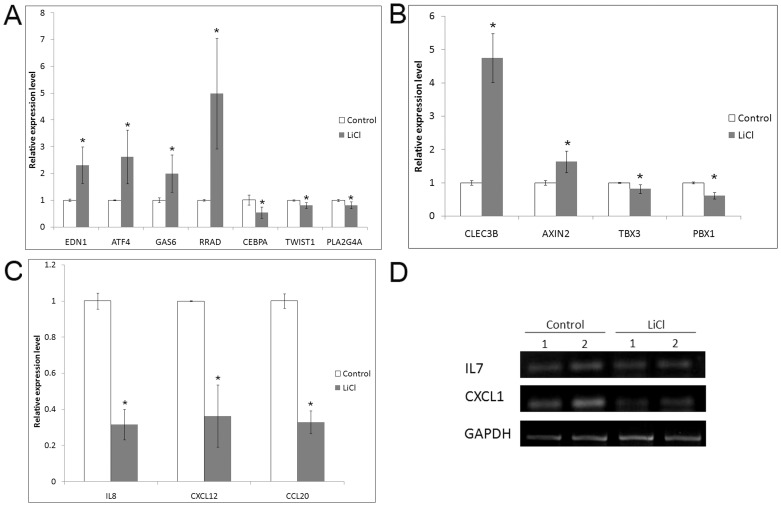
Validation of mRNA levels of genes from microarray data. (**A**) qPCR assessment of genes showing statistically significant difference on the microarray. (**B**) qPCR assessment of genes not showing statistically significant difference on the microarray. (**C**) qPCR assessment of immunogenic genes. Data of 3 independent experiments presented as mean±SD. *p≤0.05. (**D**) RT-PCR assessment of immunogenic genes. Data shown from two MSC samples:1,2.

Expression of upregulated genes ATF4, EDN1, RRAD and GAS6, and downregulated genes PLA2G4A, CEBPA and TWIST1 was validated by quantitative real time PCR (Figure 3A) and was in accordance with the microarray data. Further, we also confirmed expression of few genes which did not qualify selection criteria but are of relevance to the current study such as PBX1 (-1.4 fold, p = 0.078), TBX3 (-1.32 fold, p = 0.097), CLEC3B (+3.89 fold, p = 0.088) and AXIN2 (+1.56 fold, p = 0.285) (Figure 3B). The downregulation of osteoclastogenic and immune regulatory genes (CXCL1, CXCL12, IL7, IL8, CCL20) was verified by qPCR and PCR (Figure 3C, 3D).

### Gene ontology analysis

Further evaluation of biological processes and functions affected by LiCl using DAVID bioinformatics resources identified upregulated genes to be functionally involved in G-protein coupled receptor activity (olfactory receptors, oxytocin receptor) and signal transduction (GCOM1, CD36, IL7R, PAG1), whereas downregulated genes were enriched for cytokine, chemokine and growth factor activities (FGF7, IL7, IL8, HGF, CXCL1, CXCL12) (Table 1). Gene ontology-biological process classification also revealed overrepresentation of genes belonging to signal transduction and G-protein coupled receptor signaling as well as those implicated in mesenchymal cell differentiation (endothelin 1, neuregulin 1, cytochrome P450) among the upregulated genes. Downregulated genes were involved in organismal and system development, immune response (CFI, CXCL1, CXCL12, IL7, IL8, CCL20, CD86) and lipid biosynthesis (PTGS2, PLD1, P2RX1, KGFLP1, LASS1) (Table S4).

**Table 1:** Molecular Function Classification Of Significantly Regulated Genes.

GO ID	Term	No. of genes in our list	Total no. of genes	p Value
UPREGULATED GENES				
0004930	G-protein coupled receptor activity	9	873	0.007
0004888	transmembrane receptor activity	11	1305	0.009
0004984	olfactory receptor activity	6	431	0.013
0004871	signal transducer activity	14	2270	0.030
0060089	molecular transducer activity	14	2270	0.030
0004872	receptor activity	12	1838	0.035
DOWNREGULATED GENES				
0008083	growth factor activity	10	161	0.000
0005125	cytokine activity	8	195	0.002
0004872	receptor activity	30	1838	0.003
0046982	protein heterodimerization activity	8	208	0.003
0005102	receptor binding	17	886	0.008
0008009	chemokine activity	4	46	0.009
0042379	chemokine receptor binding	4	49	0.011
0050840	extracellular matrix binding	3	27	0.026
0060089	molecular transducer activity	31	2270	0.028
0004871	signal transducer activity	31	2270	0.028
0005111	type 2 fibroblast growth factor receptor binding	2	3	0.028
0005230	extracellular ligand-gated ion channel activity	4	72	0.030
0008330	protein tyrosine/threonine phosphatase activity	2	4	0.037

### In silico analysis of signaling pathways affected by LiCl

As a large fraction of altered genes are known to be involved in signal transduction, we performed signaling pathway analysis using PathwayMiner tool. Only 76 genes of differentially regulated genes associated with a signaling or metabolic pathway. Lithium modulated genes belonging to broad spectrum of cellular processes such as MAPK signaling (ATF4, RRAS2, DUSP2, DUSP4, ELK4, FLNC), calcium signaling (P2RX1, HTR7, OXTR, PRKACG, GRPR), FGF signaling (FGF7), JAK-STAT signaling (PIK3R5, IL7R), Wnt signaling (HNF1A, TLE4, PRKACG) and cytokine signaling (HGF, CXCL12, CXCL1, CCL20, VEGFA, IL7), as well as metabolic pathways such as glucose metabolism (RRAS2, ALDOB, HK3, PIK3R5), prostaglandin synthesis (PTGS2, HSD11B1, PLA2G4A) and arachidonic acid metabolism (PTGS2, PLA2G4A, CYP4F2). However, to identify significantly enriched pathways we used fisher exact p-value≤0.05. Only tight junction (KEGG) and smooth muscle contraction pathway (GenMAPP) were identified as the exclusive pathways being upregulated (Table S5). However, prostaglandin synthesis regulation (GenMAPP), arachidonic acid metabolism (KEGG), complement cascade (KEGG) and JAK-STAT signaling (KEGG) were a few pathways significantly downregulated.

### Lithium responsiveness of modulated osteogenic genes

**Figure 4:**
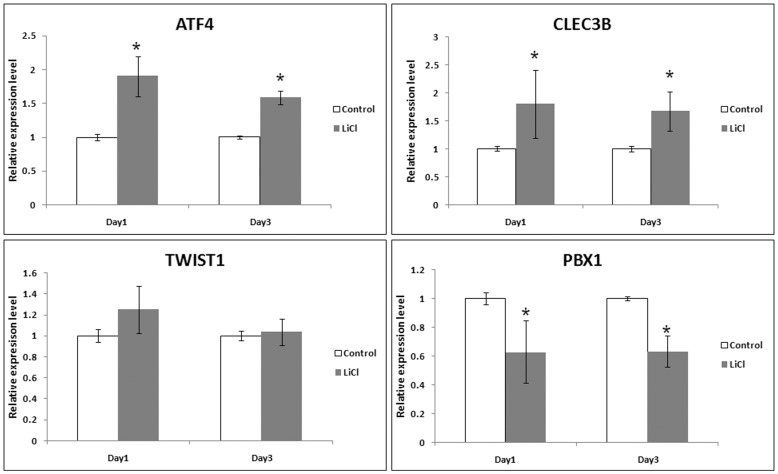
Time-dependent assessment of mRNA levels of osteogenic genes (ATF4, CLEC3B, TWIST1 and PBX1) on lithium treatment. Data of 3 independent experiments presented as mean±SD. *p≤0.05.

To better understand the responsiveness of modulated osteogenic genes (ATF4, TWIST1, PBX1, CLEC3B) to lithium, we analysed their expression following 24 h and 72 h of lithium stimulation under expansion conditions (Figure 4). ATF4 and CLEC3B were significantly induced while PBX1 was suppressed after 24 h and the effect was maintained on day 3. However, TWIST1 expression was not significantly affect at these early time points.

### Collagen I synthesis induced by lithium

**Figure 5:**
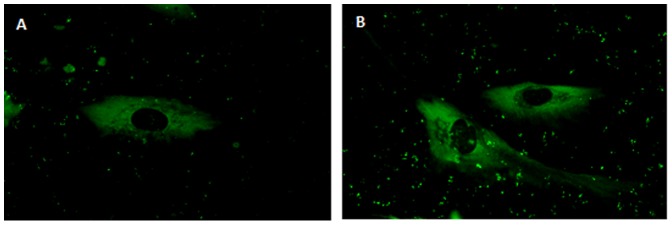
Collagen immunostaining of lithium primed hMSCs. (**A**) Control cells. (**B**) Lithium treated cells. Collagen staining in green (FITC).

Increased ATF4 expression prompted us to visualize collagen I levels in hMSC treated with lithium for 7 days under non-osteogenic conditions. Immunostaining for collagen revealed greater protein synthesis in treated cells compared to control (Figure 5).

### Enhanced osteogenic potential of lithium primed hMSCs

**Figure 6:**
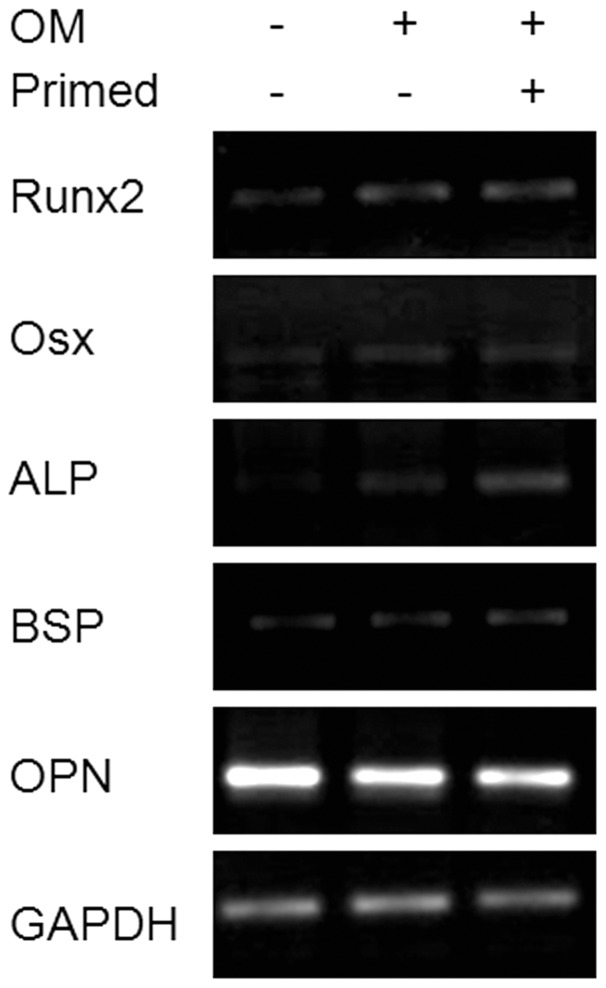
Osteogenic gene expression upon induction of differentiation in primed hMSCs. RT-PCR assessment of osteogenic marker genes. GAPDH used as endogenous control. OM: osteogenic medium, primed: lithium primed MSCs, OPN: osteopontin, GAPDH: glyceradehyde 3-phosphate dehydrogenase.

Primed hMSCs (5 mM LiCl, 7 days) were subjected to osteogenic supplements to assess the effect on osteoblast marker genes. Expression of RUNX2, ALP and bone sialoprotein (BSP) was greater in primed hMSCs upon osteogenic induction for 3 days while osteopontin (OPN) was suppressed and Osterix (OSX) was unaffected (Figure 6).

### Knockdown of ATF4, CLEC3B and RRAD reduces lithium’s effect

**Figure 7:**
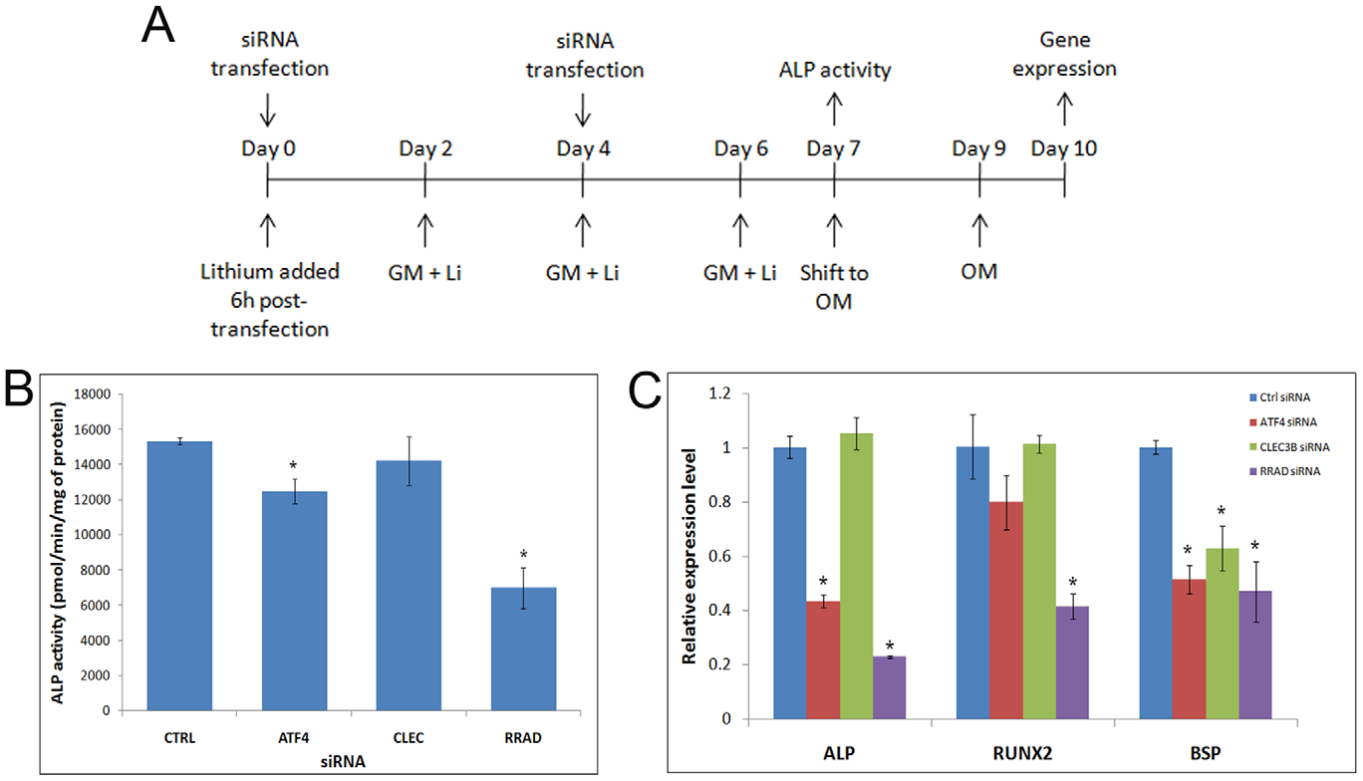
RNAi-mediated abrogation of lithium-induced priming. (**A**) Schematic representation of study plan. Following siRNA transfection of hMSCs, lithium (5 mM) was added to the cells after 6 h and the medium (containing lithium) changed every alternate day. A second round of transfection was performed on day 4 and the cells assessed for ALP actitivity on day7. For gene expression study, cells were switched to osteogenic medium (OM) on day 7 and RNA isolated after 3 days of osteogenic induction). (**B**) ALP activity upon knockdown of ATF4, CLEC3B and RRAD in primed hMSCs. Data from one MSC sample representative of other donor is presented as mean±SD. *p≤0.05 (**C**) Gene expression of osteogenic markers upon silencing of ATF4, CLEC3B and RRAD in primed hMSCs followed by osteogenic induction. Data from 2 donors presented as mean±SD. *p≤0.05.

To gain an insight into the mechanism of lithium’s action and the relevance of regulated genes identified by microarray, we employed siRNA to knockdown the expression of ATF4, CLEC3B and RRAD (study scheme shown in Figure 7A). Although ATF4 and CLEC3B have been implicated in late osteogenic differentiation, we wanted to study their effect on early osteoblastic events as is the focus of this study. The use of siRNAs resulted in downregulation of target gene expression by 40–90% on the day of analysis (data not shown). ATF4 and RRAD siRNAs suppressed lithium-induced ALP activity by 20% and 50%, respectively (Figure 7B). Osteogenic induction of lithium primed and siRNA transfected hMSCs revealed significant downregulation of ALP expression by knockdown of ATF4 and RRAD. Runx2 expression was also decreased by more than 50% by knocking down RRAD. However, BSP expression was significantly suppressed by all 3 siRNAs (Figure 7C), highlighting the role of ATF4, CLEC3B and RRAD is mediating lithium’s action.

## Discussion

Mesenchymal stem cell, the natural precursor of bone cell, is a good model for studying the osteogenic differentiation program and for the development of cell-based therapy for orthopedic defects. Although clinical trials using MSCs are being carried out, achieving directed differentiation is important to maximize their therapeutic benefit as these cells are multipotent in character [1]. We were thus interested in deciphering the molecular response of hMSCs treated with lithium for the following reasons: (1) animal model studies have demonstrated promotion of osteogenesis on lithium administration [8], [9], [34], [35], (2) data on bone quality of manic patients on lithium therapy is controversial [10]–[12], [36], and (3) in vitro studies on human MSCs employing lithium have also reported contradictory results [37]–[39].

Our results show suppression of hMSC proliferation upon lithium treatment in a concentration-dependent manner. Although lithium concentration of less than 5 mM has been shown to promote proliferation [37], [40], the observed difference could be due to differences in cell density which results in the effective number of lithium ions encountered per cell being higher under our experimental condition. However, the inhibitory effect of higher concentration is in accordance with previous reports [37]–[39]. This proliferation suppression results from the activation of Wnt signaling [41] and inhibition of GSK-3β [39], [42].

Evaluation of lithium’s effect on osteogenic differentiation ability of hMSCs using ALP as a marker revealed an increase in ALP activity at 5 mM while higher concentrations were inhibitory. The stimulatory effect of 5 mM LiCl on ALP is in contrast to earlier studies in hMSCs which reported higher concentrations to induce ALP expression [37], [38]. However, lithium is reported to induce ALP expression in mouse pluripotent cell line C3H10T1/2 [43] and enhance osteogenic differentiation of rat marrow-derived MSCs [44], but inhibit BMP-2 induced ALP expression in murine MC3T3-E1 pre-osteoblasts and myogenic C2C12 cells via inhibition of Smad1/5/8 phosphorylation [45]. Although these studies highlight the osteo-inhibitory effect of lithium on pre-osteoblasts, lithium seems to have an osteoinductive effect on early pluripotent rat MSC and C3H10T1/2 cells and on hMSCs as this study suggests. Whether lithium inhibits BMP-2 signaling in hMSCs like in myogenic C2C12 cells needs to be evaluated. However, the reasons for variations in the studies are differences in experimental conditions such as cell system, cell state, expansion medium composition like use of bFGF [37] which inhibits differentiation [46], and serum percentage, MSC expansion conditions, passage number and cell density [47], [48]. The observed inhibitory effect of LiCl at higher concentration could probably be due to disturbance of cellular homeostasis. However, it was not evaluated since it is beyond the scope of the current study. Since 5 mM LiCl gave us an increase in ALP activity consistently among the donors, this concentration was chosen for gene expression profiling.

Though de Boer et al have performed gene expression profiling of hMSCs treated with 4 mM LiCl, our study provides a more comprehensive analysis of lithium transcriptome of hMSCs since we employed MSCs from 3 different donors to account for biological variability compared to a single donor and have used an exhaustive 44000 probe array in comparison to 9000 probe array [49]. Our study also differs as we analysed transcriptome following 7 days of lithium treatment as against 4 days of treatment by de Boer et al. However, we observed similar expression change for few of the genes reported by de Boer et al, but only 2 genes CXCL12 and laminin α4 turned out to be significantly downregulated (Table S3). While the importance of laminin α4 in hMSCs needs to be evaluated, CXCL12 is reported to be highly expressed by MSCs and regulate MSC growth [50]. Recently, downregulation of CXCL12 via Wnt/GSK-3β was reported [51] concomitant with an increase in ALP expression upon induction of osteogenic differentiation suggesting decrease in CXCL12 as marker for osteoblast commitment [50], [51].

A search for genes involved in MSC differentiation revealed downregulation of adipogenesis genes CMKLR1 [52], CEBPD (-0.59 fold, p = 0.03) and CEBPA suggesting suppression of adipogenic program by lithium similar to Wnt [53]. Although lithium is reported to stabilize CEBPA [54], downregulation of CEBPA on activation of Wnt signaling has also been reported earlier [55]. Whether LiCl leads to CEBPA stabilization in hMSCs via proteasome inhibition needs to be evaluated, but it did suppress CEBPA expression probably by Wnt/β-catenin pathway activation. Moreover, the expression of 11β-hydroxysteroid dehydrogenase type1 (HSD11B1), which mediates adipogenic differentiation by converting cortisone into cortisol [56], [57], was reduced while oxytocin receptor (OXTR) promoting osteogenesis in response to oxytocin [58] was increased. Since oxytocin rescues osteoporosis in mice model [59] and glucocorticoids suppress bone formation [60] resulting in osteoporosis [61], the use of lithium therapy for preventing bone loss in patients on glucocorticoids might be a useful option which can be assessed.

As adipogenic and osteogenic differentiation programs of hMSCs are proposed to counteract each other, osteogenic genes like activating transcription factor 4 (ATF4), endothelin-1, TWIST1, stanniocalcin-1, WWTR1, PTGS2, SMOC1 and ITGA2 were found to be significantly regulated. Increased expression of ATF4 [62], [63] and EDN1 [64], and decreased expression of TWIST1 [65], [66] and ITGA2 [67] have been shown to facilitate osteogenesis. While ATF4 is capable of inducing extracellular matrix protein expression and play role in mineralization [62], [63], TWIST1, a transcription factor of bHLH family, acts as switch between adipogenesis and osteogenesis [65] by inhibiting RUNX2 activity [66]. Stanniocalcin-1 (-1.5 fold, p = 0.005) plays role in mature osteoblasts by regulating Pit1 activity [68] and inhibits bone growth on overexpression [69]. SMOC1 [70], WWTR1 [71] and PTGS2 [72] (Table S2) have been shown to promote osteoblast differentiation, but the degree of their downregulation does not seem to have a significant impact on early osteogenic program since we observed an increase in ALP activity.

The transcriptome profiling data was validated by quantitative real time PCR. Even genes (CLEC3B, TBX3, PBX1, AXIN2) which did not meet the selection criteria exhibited statistically significant change in expression when assessed by qPCR. This difference arises because of the sensitivities of the two techniques and use of different statistical analysis methods.

Gene ontology analysis of the differentially regulated genes revealed modulation of expression of genes involved in signal transduction and multicellular organismal process. Thus lithium is likely to affect different signaling pathways, thereby regulating cellular fate by altering cellular functions and processes. Therefore, pathway analysis was performed. Modulation of MAPK and Calcium signaling pathways by lithium has been reported earlier in neuronal cells [73]. MAPK signaling also plays role in regulating osteoblast differentiation [74]. Upregulation of RRAS2, a Ras-like GTPase, and downregulation of DUSP2 and DUSP4 signifies activation of ERK1/2 pathway [75]–[77]. This enhanced ERK activity is essential for regulating transcription activity of RUNX2 [78]. Thus, lithium’s effects on MSC fate are mediated either directly or indirectly both at transcriptional and post-translational levels. Further, downregulation of arachidonic acid metabolism and prostaglandin synthesis pathways seems to aid in osteogenesis by reducing levels of prostaglandin E2, which has been shown to inhibit differentiation of pre-osteoblastic cell line MC3T3-E1 [79].

We also observed modulation of many genes known to be regulated by Wnt signaling in response to lithium. For instance, EDN1 (+3.7 fold), HGF (-1.63 fold), FGF-7 (-1.65 fold) and ETV1 (-1.44 fold) demonstrated same change in expression pattern as documented in Wnt3a-treated fibroblasts [80], [81]. Upregulation of Wnt targets AXIN2 (+1.56, p = 0.285) and EDN1 was confirmed by qPCR. Other known Wnt targets such as Sp5, PTGS2, IL8 and TWIST1 were downregulated under our experimental conditions, which could be either due to difference in cell system or regulation by Wnt-independent mechanism. An interesting observation was suppression of PIAS4, a SUMO E3 ligase which represses LEF1 activity by sequestering into nuclear bodies [82], and homeobox protein 1 (-1.4, p = 0.054), a HMG-box repressor that physically interacts with LEF/TCF inhibiting their DNA binding [83]. These results highlight the likely activation of Wnt signaling cascade during the early osteogenic events upon lithium treatment of hMSCs, which is in agreement with earlier studies [43], [84], and existence of crosstalk between Wnt and MAPK signaling [85].

Evaluation of regulation of transcripts for the modulated osteogenic genes (CLEC3B, ATF4, PBX1) revealed change in expression within 24 h of lithium action and is the first report on lithium responsiveness of these genes in hMSCs. Downregulation of PBX1 is important as PBX1 inhibits osteogenic differentiation by chromatin inactivation [86]. Induction of ATF4 and CLEC3B, on the other hand, promotes mineralization [63], [87]. The observed increase in collagen I synthesis in lithium pretreated hMSCs can be attributed to the upregulation of ATF4 [63]. Osteogenic induction of primed hMSCs revealed upregulation of osteoblast markers RUNX2, ALP and BSP highlighting enhanced differentiation potential of these cells. However, the significance of the genes identified by microarray during early osteoblast differentiation is not known, and therefore, the expression of ATF4, CLEC3B and RRAD were knocked down to deduce the mechanism of lithium’s priming effect. ATF4 and CLEC3B were selected to identify any role of these late acting osteoblastic genes on early osteogenic events. Also, CLEC3B has recently been proposed as molecular marker for in vivo bone forming ability of hMSCs [88]. Knocking down CLEC3B suppressed BSP expression while ATF4 knockdown decreased expression of ALP and BSP signifying their role in regulating mineralization. The GTP binding protein, RRAD, one of the most highly upregulated gene (Table S3), is a negative regulator of Rho-Rho kinase pathway [89] and inhibition of Rho-Rho kinase signaling has been reported to enhance osteoblast differentiation and bone formation [90]–[92]. RNA interference-mediated silencing of RRAD majorly diminished lithium-induced priming as observed by downregulation of ALP activity as well as ALP, RUNX2 and BSP expression, ascribing a new role to RRAD as a regulator of osteoblast differentiation and mediator of lithium’s effect on hMSCs since it is significantly induced by lithium following 24 h treatment (data not shown). This study also brings to light the existence of possible crosstalk between the canonical Wnt signaling and Rho kinase pathway during osteoblast differentiation.

The transplantation of these primed cells would likely facilitate directed differentiation towards osteoblast aiding in treatment of orthopedic defects. However, with the increasing clinical use of scaffold material like calcium phosphate, combined use of primed cells along with scaffold seems a better approach rather than the release of lithium from the scaffold material [49] due to its proliferation inhibitory effect. But these studies need to be performed in animal models since in vitro conditions cannot recapitulate in vivo situation. It is also likely that the short-term release of lithium from calcium phosphate scaffold may prime the endogenous migrating MSCs and thus enhance reparative process due to combined osteoinductive action of lithium and osteoconductive nature of calcium phosphate.

## Conclusions

In conclusion, high throughput profiling of gene response to lithium reveals reprogramming of hMSC to favour osteogenic differentiation. A fraction of the osteogenic genes identified to be modulated upon lithium treatment play role in later stages of osteoblast differentiation apart from early regulation of ALP, Runx2 and collagen I suggesting enhanced osteogenic ability of the primed hMSCs. Moreover, the study identifies a potential role for Ras-related GTPase, RRAD, in regulating osteoblast differentiation. However, elucidation of the functional role of dozens of uncharacterized genes identified needs to be undertaken along with identification of signaling pathways other than the Wnt cascade to enhance our knowledge of MSC biology.

## Supporting Information

Figure S1 **MSC characterization.** (**A**) Flow cytometry analysis of mesenchymal markers CD44, CD105 and CD106, and hematopoietic marker CD34. Solid fill denotes isotype control and solid line represents MSC data. (**B**) Osteoblast and adipocyte differentiation of hMSCs assessed by Alizarin Red S, von Kossa staining and Oil Red-O staining.
(TIF)

Table S1 **Primer sequence for Real time PCR.**
(DOC)

Table S2 **Primer sequence for PCR.**
(DOC)

Table S3 **Complete list of differentially regulated genes.**
(DOC)

Table S4 **Biological Processes Classification of Significantly Regulated Genes.**
(DOC)

Table S5 **Signaling And Metabolic Pathways Represented By Differentially Regulated Genes.**
(DOC)
