# Supplementary figures and images for: High Throughput Transcriptome Profiling of Lithium Stimulated Human Mesenchymal Stem Cells Reveals Priming towards Osteoblastic Lineage
Source: PLoS One. 2013 Jan 30;8(1):e55769. doi: 10.1371/journal.pone.0055769 (PMC3559497; doi:10.1371/journal.pone.0055769)

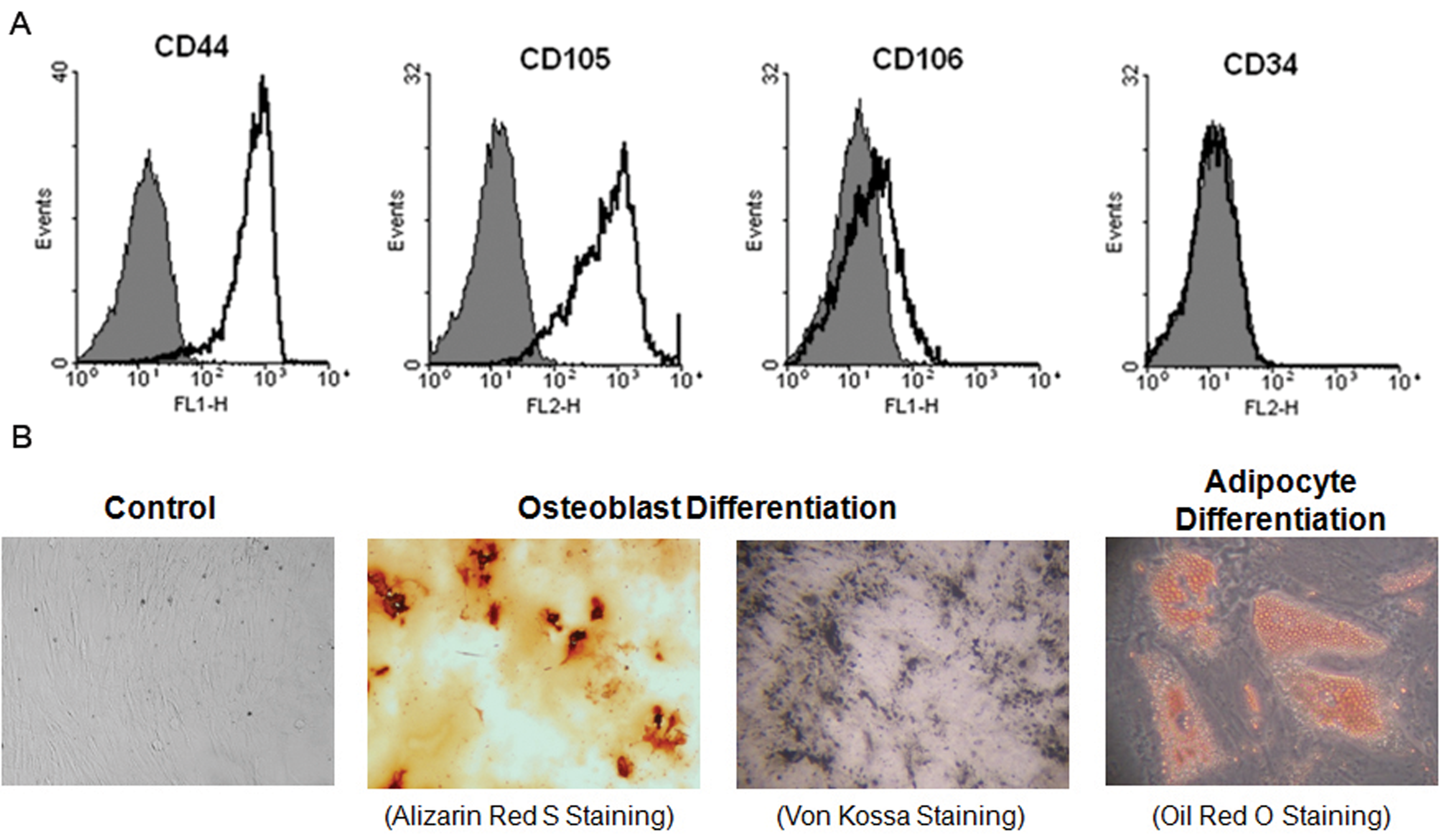

Supplement: Figure S1 — MSC characterization. (A) Flow cytometry analysis of mesenchymal markers CD44, CD105 and CD106, and hematopoietic marker CD34. Solid fill denotes isotype control and solid line represents MSC data. (B) Osteoblast and adipocyte differentiation of hMSCs assessed by Alizarin Red S, von Kossa staining and Oil Red-O staining. (TIF) [file pone.0055769.s001.tif]
