# Supplementary material for: High Throughput Transcriptome Profiling of Lithium Stimulated Human Mesenchymal Stem Cells Reveals Priming towards Osteoblastic Lineage
Source: PLoS One. 2013 Jan 30;8(1):e55769. doi: 10.1371/journal.pone.0055769 (PMC3559497; doi:10.1371/journal.pone.0055769)
Supplement: Table S1 — Primer sequence for Real time PCR. (DOC) [file pone.0055769.s002.doc]

**Table S1**: Primer sequence for Real time PCR

| **Gene** | **Primers** | **Accession no.** | **Amplicon size (bp)** | **Annealing Temp. (^o^C)** |
| --- | --- | --- | --- | --- |
| CLEC3B | Forward: 5’ CACCACCGAGCCACCAACCC 3’  Reverse: 5’ TCAGCAGGGCCACCTCCTGG 3’ | NM_003278 | 128 | 62 |
| TBX3 | Forward: 5’ AGCCGCCTCCACTGTAGGGA 3’  Reverse: 5’ TGGAGATCTTGGCCGCGTCG 3’ | NM_016569 | 125 | 62 |
| PBX1 | Forward: 5’ GCGGGAGGAAGCAGGACATTGGA 3’  Reverse: 5’ CAAGGCAGGCTTCATTCTGTGGCAG 3’ | NM_002585 | 122 | 62 |
| PLA2GA4 | Forward: 5’ AGGCCAAGTGACTCTAGTCCTCCG 3’  Reverse: 5’ AGCACTCCTTCAGCCCTTCCCG 3’ | NM_024420 | 127 | 62 |
| ATF4 | Forward: 5’ AAGGCGGGCTCCTCCGAATG 3’  Reverse: 5’ CCCAACAGGGCATCCAAGTCGA 3’ | NM_001675 | 140 | 62 |
| AXIN2 | Forward: 5’ AGGCCTGTCGCAGGCTAGCT 3’  Reverse: 5’ TCCCGTCTGAACAGTGGCCGA 3’ | NM_004655 | 104 | 62 |
| EDN1 | Forward: 5’ TTGTGGCTTGCCAAGGAGCTCC 3’  Reverse: 5’ GTGGGTTTCTCCCCGCCGTT 3’ | NM_001955 | 85 | 62 |
| TWIST1 | Forward: 5’ GGTCCATGTCCGCGTCCCAC 3’  Reverse: 5’ AATGACATCTAGGTCTCCGGCCCTG 3’ | NM_000474 | 73 | 62 |
| GAS6 | Forward: 5’ AACTCAGGCTTCGCCACCTGCG 3’  Reverse: 5’ CCCTTCCTATCGCAGGGGTTGGG 3’ | NM_000820 | 71 | 62 |
| CEBPA | Forward: 5’ ACGATCAGTCCATCCCAGAG 3’  Reverse: 5’ TTCACATTGCACAAGGCACT 3’ | NM_004364 | 122 | 62 |
| RRAD | Forward: 5’ TGAACTTGCAGTCAAAGACCA 3’  Reverse: 5’ GCAAACAGATGATGTGCCC 3’ | NM_004165 | 116 | 58 |
| ALP | Forward: 5’ GGGATAAAGCAGGTCTTGGGGTGC 3’  Reverse: 5’ CGCTTGGTCTCGCCAGTACTTGG 3’ | NM_000478 | 126 | 62 |
| RUNX2 | Forward: 5’ CCTAAATCACTGAGGCGGTC 3’  Reverse: 5’ CAGTAGATGGACCTCGGGAA 3’ | NM_001024630 | 91 | 62 |
| BSP | Forward: 5’ CAGAAAGTGTGGTATTCTCAGCC 3’  Reverse: 5’ GGGCAGTAGTGACTCATCCG 3’ | NM_004967 | 143 | 56 |
| IL8 | Forward: 5’ GAGTGGACCACACTGCGCCAA 3’  Reverse: 5’ TCCACAACCCTCTGCACCCAGTT 3’ | NM_000584 | 102 | 58 |
| CXCL12 | Forward: 5’ AACGCCAAGGTCGTGGTCGT 3’  Reverse: 5’ AGCTCAGGCTGACGGGCTTC 3’ | NM_199168 | 79 | 58 |
| CCL20 | Forward: 5’ TCCACCTCTGCGGCGAATCAGA 3’  Reverse: 5’ TCATTGGCCAGCTGCCGTGT 3’ | NM_004591 | 115 | 58 |
| 18S rRNA | Forward: 5’ CGGTACAGTGAAACTGCGAA 3’  Reverse: 5’ TCCAAGTAGGAGAGGAGCGA 3’ | NM_003286 | 75 | 58 |
