# Supplementary material for: High Throughput Transcriptome Profiling of Lithium Stimulated Human Mesenchymal Stem Cells Reveals Priming towards Osteoblastic Lineage
Source: PLoS One. 2013 Jan 30;8(1):e55769. doi: 10.1371/journal.pone.0055769 (PMC3559497; doi:10.1371/journal.pone.0055769)
Supplement: Table S2 — Primer sequence for PCR. (DOC) [file pone.0055769.s003.doc]

**Table S2**: Primer sequence for PCR

| **Gene** | **Primers** | **Accession no.** | **Amplicon size (bp)** | **Annealing Temp. (^o^C)** |
| --- | --- | --- | --- | --- |
| IL7 | Forward: 5’ TGCTGCTCGCAAGTTGAGGCAATT 3’  Reverse: 5’ CCAGGGCAGCTGGTTTTCTTCCTTT 3’ | NM_000880 | 140 | 58 |
| CXCL1 | Forward: 5’ GGCGGAAAGCTTGCCTCAATCCTG 3’  Reverse: 5’ GCCTCCTTCAGGAACAGCCACCA 3’ | NM_001511 | 129 | 58 |
| GAPDH | Forward: 5’ GTCAGTGGTGGACCTGACCT 3’  Reverse: 5’ TGTGAGGAGGGGAGATTCAG 3’ | NM_002046 | 402 | 56 |
| ALP | Forward: 5’ GGACATGCAGTACGAGCTGA 3’  Reverse: 5’ GCAGTGAAGGGCTTCTTGTC 3’ | NM_000478 | 357 | 58 |
| BSP | Forward: 5’ CTGCTTCCTCACTCCAGGAC 3’  Reverse: 5’ GTCCTCTCCATAGCCCAGTG 3’ | NM_004967 | 394 | 62 |
| RUNX2 | Forward: 5’ TCTGGCCTTCCACTCTCAGT 3’  Reverse: 5’ CACTCTGGCTTTGGGAAGAG 3’ | NM_001024630 | 252 | 58 |
| OSX | Forward: 5’ CCTGGCTCCTTGGGACCCGT 3’  Reverse: 5’ AGCCTGAGGTGGGTGCTGGA 3’ | NM_152860 | 296 | 62 |
| OPN | Forward: 5’ GCCACATGGCTAAACCCTGACCC 3’  Reverse: 5’ TTCGGTTGCTGGCAGGTCCG 3’ | NM_001040058 | 270 | 65 |
