# Supplementary material for: High Throughput Transcriptome Profiling of Lithium Stimulated Human Mesenchymal Stem Cells Reveals Priming towards Osteoblastic Lineage
Source: PLoS One. 2013 Jan 30;8(1):e55769. doi: 10.1371/journal.pone.0055769 (PMC3559497; doi:10.1371/journal.pone.0055769)
Supplement: Table S3 — Complete list of differentially regulated genes. (DOC) [file pone.0055769.s004.doc]

**Table S3: Complete list of differentially regulated genes (log_2_ fold change≥±0.6, p value≤ 0.05)**

| **Gene** | | **Accession no.** | **Description** | **Avg fold (log _2_)** | **p value** |
| --- | --- | --- | --- | --- | --- |
| **UPREGULATED GENES** | | | | | |
| LOC727982 | | BC047589 | hypothetical protein LOC727982 | **3.59** | 0.005 |
| CD36 | | NM_001001547 | CD36 molecule (thrombospondin receptor) | **3.34** | 0.000 |
| RRAD | | NM_004165 | Ras-related associated with diabetes | **3.20** | 0.003 |
| CRIP1 | | NM_001311 | cysteine-rich protein 1 (intestinal) | **2.43** | 0.049 |
| SFTA1P | | AY102069 | Surfactant associated 1 (pseudogene) | **2.30** | 0.046 |
| KISS1 | | NM_002256 | KiSS-1 metastasis-suppressor | **2.28** | 0.005 |
| FOXL2 | | NM_023067 | forkhead box L2 | **2.26** | 0.001 |
| TPPP3 | | NM_016140 | tubulin polymerization-promoting protein family member 3 | **2.00** | 0.024 |
| PRR15 | | NM_175887 | proline rich 15 | **1.90** | 0.000 |
| EDN1 | | NM_001955 | endothelin 1 | **1.89** | 0.001 |
| FAM46B | | NM_052943 | family with sequence similarity 46, member B | **1.72** | 0.021 |
| CD70 | | NM_001252 | CD70 molecule | **1.71** | 0.026 |
| C13orf15 | | NM_014059 | chromosome 13 open reading frame 15 | **1.70** | 0.020 |
| C3orf51 | | U88965 | chromosome 3 open reading frame 51 | **1.65** | 0.012 |
| GRPR | | NM_005314 | gastrin-releasing peptide receptor | **1.64** | 0.018 |
|  | | ENST00000367073 | Unknown | **1.63** | 0.036 |
| GIPC2 | | NM_017655 | GIPC PDZ domain containing family, member 2 | **1.61** | 0.002 |
| NRG1 | | NM_013962 | neuregulin 1 | **1.57** | 0.003 |
| ELK4 | | NM_001973 | ELK4, ETS-domain protein (SRF accessory protein 1) | **1.56** | 0.020 |
|  | | AK022678 | hypothetical LOC196707 | **1.53** | 0.047 |
| OR51B5 | | NM_001005567 | olfactory receptor, family 51, subfamily B, member 5 | **1.53** | 0.011 |
| OR9K2 | | NM_001005243 | olfactory receptor, family 9, subfamily K, member 2 | **1.50** | 0.002 |
| TAS2R9 | | NM_023917 | taste receptor, type 2, member 9 | **1.50** | 0.006 |
| OR9A2 | | NM_001001658 | olfactory receptor, family 9, subfamily A, member 2 | **1.47** | 0.011 |
| LSAMP | | NM_002338 | limbic system-associated membrane protein | **1.46** | 0.029 |
| SH2D5 | | AK124869 | SH2 domain containing 5 | **1.43** | 0.007 |
|  | | NM_001013617 | hypothetical protein LOC541469 | **1.43** | 0.029 |
| SPNS3 | | NM_182538 | spinster homolog 3 (Drosophila) | **1.42** | 0.024 |
| SLC7A14 | | AK094547 | hypothetical protein FLJ37228 | **1.41** | 0.034 |
| GLT25D2 | | NM_015101 | glycosyltransferase 25 domain containing 2 | **1.40** | 0.016 |
| OR10H1 | | NM_013940 | olfactory receptor, family 10, subfamily H, member 1 | **1.37** | 0.021 |
|  | | CR740121 | Transcribed locus | **1.37** | 0.045 |
| A1BG | | NM_130786 | alpha-1-B glycoprotein | **1.37** | 0.038 |
| OXTR | | NM_000916 | oxytocin receptor | **1.35** | 0.003 |
| RPS20 | | NM_001146227 | ribosomal protein S20 | **1.34** | 0.020 |
| DIRC3 | | NR_026597 | disrupted in renal carcinoma 3 | **1.32** | 0.020 |
| SERPINB7 | | NM_003784 | serpin peptidase inhibitor, clade B (ovalbumin), member 7 | **1.31** | 0.009 |
| MLLT4 | | NM_005936 | myeloid/lymphoid or mixed-lineage leukemia (trithorax homolog, Drosophila); translocated to, 4 | **1.28** | 0.022 |
| CYP26C1 | | NM_183374 | cytochrome P450, family 26, subfamily C, polypeptide 1 | **1.28** | 0.017 |
| LCN10 | | NM_001001712 | lipocalin 10 | **1.27** | 0.019 |
|  | | ENST00000390550 | Unknown | **1.26** | 0.047 |
| FAM43B | | NM_207334 | family with sequence similarity 43, member B | **1.26** | 0.042 |
| LRRC32 | | NM_005512 | leucine rich repeat containing 32 | **1.23** | 0.006 |
|  | | ENST00000271847 | Unknown | **1.22** | 0.042 |
|  | | DQ680071 | Mir-223 transcript variant 1 mRNA, complete sequence | **1.21** | 0.031 |
| RAB11FIP1 | | NM_001002233 | RAB11 family interacting protein 1 (class I) | **1.21** | 0.034 |
| IL7R | | NM_002185 | interleukin 7 receptor | **1.20** | 0.012 |
| SPATA18 | | NM_145263 | spermatogenesis associated 18 homolog (rat) | **1.20** | 0.039 |
|  | | AK124776 | CDNA FLJ42786 fis, clone BRAWH3006761 | **1.20** | 0.038 |
| STXBP6 | | NM_014178 | syntaxin binding protein 6 (amisyn) | **1.20** | 0.032 |
| ALDOB | | NM_000035 | aldolase B, fructose-bisphosphate | **1.18** | 0.039 |
| OR52E4 | | NM_001005165 | olfactory receptor, family 52, subfamily E, member 4 | **1.17** | 0.013 |
| PSG1 | | NM_006905 | pregnancy specific beta-1-glycoprotein 1 | **1.17** | 0.038 |
| CHAT | | NM_020549 | choline acetyltransferase | **1.14** | 0.023 |
| RASSF3 | | NM_178169 | Ras association (RalGDS/AF-6) domain family 3 | **1.14** | 0.017 |
| PSG8 | | NM_182707 | pregnancy specific beta-1-glycoprotein 8 | **1.14** | 0.031 |
| PSG6 | | NM_002782 | pregnancy specific beta-1-glycoprotein 6 | **1.14** | 0.035 |
| GCOM1 | | NM_152451 | GRINL1A complex locus | **1.12** | 0.040 |
| NT5DC3 | | NM_016575 | 5'-nucleotidase domain containing 3 | **1.11** | 0.048 |
| PSG5 | | ENST00000342951 | pregnancy specific beta-1-glycoprotein 5 | **1.10** | 0.029 |
| ZDHHC19 | | NM_001039617 | zinc finger, DHHC-type containing 19 | **1.08** | 0.042 |
| RAB3B | | ENST00000371655 | RAB3B, member RAS oncogene family | **1.07** | 0.020 |
| TLE4 | | NM_007005 | transducin-like enhancer of split 4 (E(sp1) homolog, Drosophila) | **1.06** | 0.027 |
|  | | NM_207462 | FLJ45684 locus | **1.06** | 0.027 |
| OR5M11 | | NM_001005245 | olfactory receptor, family 5, subfamily M, member 11 | **1.05** | 0.035 |
| PAG1 | | NM_018440 | phosphoprotein associated with glycosphingolipid microdomains 1 | **1.05** | 0.034 |
| PSG2 | | NM_031246 | pregnancy specific beta-1-glycoprotein 2 | **1.05** | 0.026 |
|  | | AF086468 | Full length insert cDNA clone ZD86H05 | **1.04** | 0.011 |
|  | | XR_016161 | keratin 18 pseudogene 23 | **1.03** | 0.017 |
| CCDC81 | | NM_021827 | coiled-coil domain containing 81 | **1.03** | 0.039 |
| LAYN | | NM_178834 | Layilin | **1.03** | 0.001 |
|  | | AL359605 | CDNA FLJ39162 fis, clone OCBBF2002376 | **1.02** | 0.015 |
| KRT18P33 | | XR_019330 | similar to Keratin, type I cytoskeletal 18 | **1.01** | 0.044 |
|  | | BC041955 | CDNA clone IMAGE:5301910 | **1.01** | 0.035 |
| KRT18 | | NM_000224 | keratin 18 | **1.00** | 0.030 |
| FLJ40504 | | NM_173624 | hypothetical protein FLJ40504 | **0.99** | 0.021 |
|  | | BX647685 | MRNA; cDNA DKFZp686J0581 | **0.98** | 0.010 |
|  | | XR_016695 | keratin 18 pseudogene 41 | **0.98** | 0.016 |
| NUPR1 | | NM_012385 | nuclear protein 1 | **0.94** | 0.006 |
| PDLIM1 | | NM_020992 | PDZ and LIM domain 1 (elfin) | **0.94** | 0.028 |
| CRIP2 | | NM_001312 | cysteine-rich protein 2 | **0.93** | 0.035 |
| LOC284454 | | BX640708 | hypothetical protein LOC284454 | **0.93** | 0.032 |
| C7orf52 | | NM_198571 | chromosome 7 open reading frame 52 | **0.93** | 0.039 |
| GAS6 | | NM_000820 | growth arrest-specific 6 | **0.92** | 0.025 |
| KIRREL3 | | ENST00000278934 | kin of IRRE like 3 (Drosophila) | **0.90** | 0.018 |
| ARHGDIB | | NM_001175 | Rho GDP dissociation inhibitor (GDI) beta | **0.86** | 0.032 |
| MPP5 | | NM_022474 | membrane protein, palmitoylated 5 (MAGUK p55 subfamily member 5) | **0.84** | 0.026 |
| FLNC | | NM_001458 | filamin C, gamma (actin binding protein 280) | **0.83** | 0.011 |
| ATF4 | | NM_001675 | activating transcription factor 4 (tax-responsive enhancer element B67) | **0.83** | 0.015 |
| LOC643894 | | XR_016475 | similar to Cyclic AMP-dependent transcription factor ATF-4 (Activating transcription factor 4) | **0.82** | 0.027 |
|  | | AF075027 | Full length insert cDNA YI37C01 | **0.79** | 0.012 |
| RRAS2 | | NM_012250 | related RAS viral (r-ras) oncogene homolog 2 | **0.62** | 0.041 |
| GAS5 | | NR_002578 | growth arrest-specific 5 | **0.62** | 0.048 |
| **DOWNREGULATED GENES** | | | | | |
| IL8 | NM_000584 | | interleukin 8 | **-2.42** | 0.002 |
|  | THC2559929 | | Unknown | **-2.18** | 0.000 |
|  | XR_015921 | | hypothetical protein LOC729279 | **-2.16** | 0.000 |
|  | XR_015536 | | hypothetical protein LOC731599 | **-2.14** | 0.000 |
| PIAS4 | ENST00000262971 | | protein inhibitor of activated STAT, 4 | **-2.09** | 0.000 |
|  | AK130207 | | similar to eukaryotic translation elongation factor 1 delta isoform 1 | **-2.07** | 0.000 |
| ANGPT2 | NM_001147 | | angiopoietin 2 | **-1.96** | 0.001 |
|  | XR_019110 | | similar to ribosomal protein L31 | **-1.96** | 0.002 |
| LOC731656 | NR_027454 | | hypothetical LOC731656 | **-1.95** | 0.006 |
| SPOCK2 | NM_014767 | | sparc/osteonectin, cwcv and kazal-like domains proteoglycan (testican) 2 | **-1.91** | 0.001 |
|  | XR_019587 | | similar to ATP synthase B chain, mitochondrial precursor | **-1.90** | 0.001 |
| THBD | NM_000361 | | Thrombomodulin | **-1.85** | 0.016 |
| PARP4 | NM_006437 | | poly (ADP-ribose) polymerase family, member 4 | **-1.85** | 0.004 |
| ZMAT4 | NM_024645 | | zinc finger, matrin type 4 | **-1.84** | 0.012 |
|  | XM_001127738 | | similar to CoLlagen sequence X-hybridizing family member (clx-1) | **-1.82** | 0.000 |
|  | AK130118 | | CDNA FLJ26608 fis, clone LVR00914 | **-1.78** | 0.030 |
| COL22A1 | NM_152888 | | collagen, type XXII, alpha 1 | **-1.77** | 0.038 |
| PRSS1 | NM_002769 | | protease, serine, 1 (trypsin 1) | **-1.72** | 0.037 |
|  | THC2687779 | | Unknown | **-1.72** | 0.006 |
| ST13 | NM_003932 | | suppression of tumorigenicity 13 (colon carcinoma) (Hsp70 interacting protein) | **-1.70** | 0.003 |
| BPNT1 | NM_006085 | | 3'(2'), 5'-bisphosphate nucleotidase 1 | **-1.70** | 0.004 |
| CSGALNACT1 | NM_018371 | | chondroitin beta1,4 N-acetylgalactosaminyltransferase | **-1.68** | 0.043 |
|  | THC2732364 | | Unknown | **-1.67** | 0.004 |
| BZW1P1 | XR_017648 | | similar to basic leucine zipper and W2 domains 1 | **-1.67** | 0.011 |
|  | XR_019557 | | hypothetical gene supported by NM_014886 | **-1.67** | 0.001 |
| YPEL4 | NM_145008 | | yippee-like 4 (Drosophila) | **-1.65** | 0.011 |
| UPP1 | NM_181597 | | uridine phosphorylase 1 | **-1.64** | 0.037 |
|  | XR_016825 | | hypothetical LOC392352 | **-1.63** | 0.006 |
|  | ENST00000278654 | | Unknown | **-1.62** | 0.003 |
| GOLGA8E | NM_001012423 | | golgi autoantigen, golgin subfamily a, 8E | **-1.60** | 0.003 |
| ICMT | NM_012405 | | isoprenylcysteine carboxyl methyltransferase | **-1.57** | 0.006 |
| FLJ13197 | AK023259 | | hypothetical FLJ13197 | **-1.56** | 0.016 |
|  | NR_002768 | | hydatidiform mole associated and imprinted (non-protein coding) | **-1.56** | 0.003 |
| HSD11B1 | NM_181755 | | hydroxysteroid (11-beta) dehydrogenase 1 | **-1.55** | 0.003 |
| PLXNA4 | NM_001105543 | | plexin A4 | **-1.54** | 0.024 |
|  | NR_002834 | | dual specificity phosphatase 5 pseudogene | **-1.54** | 0.005 |
| NHLRC2 | BC032598 | | NHL repeat containing 2 | **-1.52** | 0.006 |
|  | BM547196 | | Unknown | **-1.51** | 0.000 |
| FGF7 | NM_002009 | | fibroblast growth factor 7 (keratinocyte growth factor) | **-1.50** | 0.007 |
| LOC145694 | AK056793 | | hypothetical protein LOC145694 | **-1.48** | 0.003 |
|  | XM_070233 | | similar to ribosomal protein L10a | **-1.48** | 0.002 |
| SYT12 | NM_177963 | | synaptotagmin XII | **-1.47** | 0.005 |
| CYP4F2 | NM_001082 | | cytochrome P450, family 4, subfamily F, polypeptide 2 | **-1.45** | 0.000 |
| C6orf176 | NR_026860 | | chromosome 6 open reading frame 176 | **-1.44** | 0.048 |
| AGAP4 | NM_133446 | | centaurin, gamma-like family, member 1 | **-1.44** | 0.007 |
| HIST1H3J | NM_003535 | | histone cluster 1, H3j | **-1.44** | 0.002 |
|  | THC2594845 | | Unknown | **-1.43** | 0.006 |
|  | M14087 | | HL14 gene encoding beta-galactoside-binding lectin, 3' end, clone 2 | **-1.43** | 0.004 |
| PTGS2 | NM_000963 | | prostaglandin-endoperoxide synthase 2 (prostaglandin G/H synthase and cyclooxygenase) | **-1.42** | 0.000 |
| HGF | NM_001010931 | | hepatocyte growth factor | **-1.42** | 0.001 |
|  | BC041459 | | Homo sapiens, clone IMAGE:5207242, mRNA | **-1.41** | 0.010 |
| ITGA2 | NM_002203 | | integrin, alpha 2 (CD49B) | **-1.41** | 0.018 |
| UMODL1 | NM_173568 | | uromodulin-like 1 | **-1.40** | 0.015 |
| CCL20 | NM_004591 | | chemokine (C-C motif) ligand 20 | **-1.38** | 0.013 |
| PTGES | NM_004878 | | prostaglandin E synthase | **-1.38** | 0.014 |
| CXCL1 | NM_001511 | | chemokine (C-X-C motif) ligand 1 (melanoma growth stimulating activity, alpha) | **-1.38** | 0.000 |
| KYNU | NM_003937 | | kynureninase (L-kynurenine hydrolase) | **-1.38** | 0.006 |
| CACNG6 | NM_145814 | | calcium channel, voltage-dependent, gamma subunit 6 | **-1.36** | 0.026 |
|  | THC2668815 | | Unknown | **-1.35** | 0.003 |
| SLC14A1 | NM_015865 | | solute carrier family 14 (urea transporter), member 1 (Kidd blood group) | **-1.35** | 0.014 |
|  | XR_018240 | | similar to nucleophosmin 1 isoform 1 | **-1.35** | 0.003 |
| AREG | NM_001657 | | amphiregulin (schwannoma-derived growth factor) | **-1.34** | 0.017 |
|  | THC2503300 | | Unknown | **-1.33** | 0.049 |
| TWIST1 | NM_000474 | | twist homolog 1 (acrocephalosyndactyly 3; Saethre-Chotzen syndrome) (Drosophila) | **-1.31** | 0.030 |
| FAIM2 | NM_012306 | | Fas apoptotic inhibitory molecule 2 | **-1.30** | 0.007 |
| ANXA10 | NM_007193 | | annexin A10 | **-1.30** | 0.012 |
| RNF113B | NM_178861 | | ring finger protein 113B | **-1.29** | 0.003 |
| MIA | NM_006533 | | melanoma inhibitory activity | **-1.29** | 0.007 |
|  | THC2633920 | | Unknown | **-1.29** | 0.004 |
| CEBPA | NM_004364 | | CCAAT/enhancer binding protein (C/EBP), alpha | **-1.28** | 0.013 |
| DIO3OS | AF305836 | | deiodinase, iodothyronine, type III opposite strand | **-1.28** | 0.009 |
| LCE3E | NM_178435 | | late cornified envelope 3E | **-1.28** | 0.001 |
|  | ENST00000324961 | | Unknown | **-1.27** | 0.005 |
| RBMS1 | NM_002897 | | RNA binding motif, single stranded interacting protein 1 | **-1.25** | 0.023 |
|  | ENST00000355095 | | Unknown | **-1.25** | 0.009 |
| CMKLR1 | AK126405 | | Chemokine-like receptor 1 | **-1.24** | 0.033 |
|  | THC2676548 | | Unknown | **-1.24** | 0.007 |
|  | AK125162 | | CDNA FLJ43172 fis, clone FCBBF3007242 | **-1.23** | 0.042 |
| AMY2B | AY129015 | | Amylase, alpha 2B (pancreatic) | **-1.23** | 0.008 |
| PECAM1 | NM_000442 | | platelet/endothelial cell adhesion molecule | **-1.23** | 0.005 |
| TFPI2 | ENST00000222543 | | tissue factor pathway inhibitor 2 | **-1.23** | 0.019 |
| SP5 | NM_001003845 | | Sp5 transcription factor | **-1.23** | 0.002 |
|  | XR_019042 | | similar to RIKEN cDNA 0610013E23 | **-1.22** | 0.008 |
| ATXN8OS | NR_002717 | | ATXN8 opposite strand (non-protein coding) | **-1.22** | 0.014 |
| SULT1A2 | NM_177528 | | sulfotransferase family, cytosolic, 1A, phenol-preferring, member 2 | **-1.21** | 0.007 |
| NR4A2 | NM_006186 | | nuclear receptor subfamily 4, group A, member 2 | **-1.20** | 0.006 |
|  | NR_024569 | | hypothetical LOC100130872 | **-1.19** | 0.008 |
| HERC3 | NM_014606 | | hect domain and RLD 3 | **-1.19** | 0.007 |
| GALNTL2 | NM_054110 | | UDP-N-acetyl-alpha-D-galactosamine:polypeptide N-acetylgalactosaminyltransferase-like 2 | **-1.19** | 0.011 |
|  | XR_018851 | | similar to phosphoglycerate mutase 1 | **-1.18** | 0.029 |
| OR2B11 | NM_001004492 | | olfactory receptor, family 2, subfamily B, member 11 | **-1.18** | 0.006 |
| DMKN | NM_033317 | | Dermokine | **-1.18** | 0.008 |
| SLC22A3 | NM_021977 | | solute carrier family 22 (extraneuronal monoamine transporter), member 3 | **-1.17** | 0.011 |
| DNAH3 | AK027211 | | Dynein, axonemal, heavy chain 3 | **-1.16** | 0.002 |
| CARD17 | NM_001007232 | | inhibitory caspase recruitment domain (CARD) protein | **-1.16** | 0.020 |
| SPPL2B | NM_001077238 | | signal peptide peptidase-like 2B | **-1.15** | 0.002 |
| SMOC1 | NM_022137 | | SPARC related modular calcium binding 1 | **-1.15** | 0.003 |
| GPR115 | NM_153838 | | G protein-coupled receptor 115 | **-1.14** | 0.031 |
| FBRSL1 | AF490258 | | KIAA1545 protein | **-1.14** | 0.002 |
| OR2W1 | NM_030903 | | olfactory receptor, family 2, subfamily W, member 1 | **-1.14** | 0.015 |
| LCE2D | NM_178430 | | late cornified envelope 2D | **-1.13** | 0.016 |
|  | XM_928198 | | similar to 60S ribosomal protein L12 | **-1.13** | 0.002 |
| CAMK2N2 | NM_033259 | | calcium/calmodulin-dependent protein kinase II inhibitor 2 | **-1.12** | 0.019 |
| NR4A1 | NM_002135 | | nuclear receptor subfamily 4, group A, member 1 | **-1.12** | 0.009 |
| CFI | NM_000204 | | complement factor I | **-1.10** | 0.010 |
| TXNDC2 | NM_032243 | | thioredoxin domain containing 2 (spermatozoa) | **-1.10** | 0.023 |
| FLJ40330 | BC031698 | | similar to protein immuno-reactive with anti-PTH polyclonal antibodies | **-1.10** | 0.018 |
| FBXO36 | NM_174899 | | F-box protein 36 | **-1.09** | 0.041 |
|  | AF119858 | | epidermal growth factor receptor pathway substrate 15-like 2 | **-1.09** | 0.013 |
| HTR7 | NM_019859 | | 5-hydroxytryptamine (serotonin) receptor 7 (adenylate cyclase-coupled) | **-1.08** | 0.004 |
|  | XR_018322 | | similar to adaptor-related protein complex 2, beta 1 subunit | **-1.08** | 0.022 |
|  | BU191598 | | Transcribed locus | **-1.08** | 0.005 |
| SLC2A3 | NM_006931 | | solute carrier family 2 (facilitated glucose transporter), member 3 | **-1.08** | 0.017 |
|  | ENST00000314123 | | Unknown | **-1.08** | 0.023 |
| NCRNA00153 | BC039296 | | chromosome 20 open reading frame 19 | **-1.07** | 0.011 |
| RXRG | NM_001009598 | | retinoid X receptor, gamma | **-1.07** | 0.032 |
| EGR3 | NM_004430 | | early growth response 3 | **-1.07** | 0.003 |
| ISCA1 | NM_030940 | | iron-sulfur cluster assembly 1 homolog (S. cerevisiae) | **-1.07** | 0.016 |
|  | ENST00000369572 | | Unknown | **-1.07** | 0.009 |
| FLJ39609 | NR_026874 | | similar to hCG1995469 | **-1.06** | 0.032 |
|  | CD556746 | | Transcribed locus | **-1.06** | 0.010 |
| CD86 | NM_006889 | | CD86 molecule | **-1.06** | 0.010 |
| NCOA7 | NM_181782 | | nuclear receptor coactivator 7 | **-1.05** | 0.011 |
|  | BC035260 | | Unknown | **-1.05** | 0.046 |
| CXCL12 | NM_199168 | | chemokine (C-X-C motif) ligand 12 (stromal cell-derived factor 1) | **-1.05** | 0.013 |
| CPN2 | NM_001080513 | | carboxypeptidase N, polypeptide 2 | **-1.04** | 0.012 |
| HHIPL2 | NM_024746 | | KIAA1822-like | **-1.04** | 0.026 |
| LASS1 | NM_001492 | | growth differentiation factor 1 | **-1.04** | 0.025 |
|  | ENST00000312015 | | Unknown | **-1.04** | 0.013 |
| INE1 | BC069772 | | inactivation escape 1 | **-1.03** | 0.017 |
|  | BC013657 | | CDNA clone IMAGE:4152983 | **-1.03** | 0.033 |
| STC1 | NM_003155 | | stanniocalcin 1 | **-1.03** | 0.005 |
|  | BM129308 | | Transcribed locus | **-1.02** | 0.015 |
|  | THC2579650 | | Unknown | **-1.02** | 0.022 |
| ERRFI1 | NM_018948 | | ERBB receptor feedback inhibitor 1 | **-1.02** | 0.002 |
| MLLT3 | NM_004529 | | myeloid/lymphoid or mixed-lineage leukemia (trithorax homolog, Drosophila); translocated to, 3 | **-1.02** | 0.011 |
| AGPAT9 | NM_032717 | | lung cancer metastasis-associated protein | **-1.01** | 0.046 |
| PDE4D | NM_006203 | | phosphodiesterase 4D, cAMP-specific | **-1.01** | 0.035 |
| RAB27B | NM_004163 | | RAB27B, member RAS oncogene family | **-1.01** | 0.026 |
|  | AK123446 | | CDNA FLJ41452 fis, clone BRSTN2010363 | **-1.01** | 0.006 |
| PLD1 | AK026225 | | Phospholipase D1, phosphatidylcholine-specific | **-1.00** | 0.017 |
| RASD1 | NM_016084 | | RAS, dexamethasone-induced 1 | **-1.00** | 0.038 |
| CAND1 | AF157326 | | cullin-associated and neddylation-dissociated 1 | **-1.00** | 0.023 |
|  | ENST00000309901 | | Unknown | **-1.00** | 0.016 |
|  | XR_019598 | | similar to Chloride intracellular channel protein 1 | **-1.00** | 0.003 |
|  | AK024924 | | CDNA: FLJ21271 fis, clone COL01751 | **-0.99** | 0.012 |
|  | THC2609820 | | Unknown | **-0.99** | 0.026 |
| CYGB | NM_134268 | | Cytoglobin | **-0.99** | 0.021 |
| CLDN20 | NM_001001346 | | claudin 20 | **-0.99** | 0.015 |
| TPRXL | NR_002223 | | tetra-peptide repeat homeobox-like | **-0.99** | 0.019 |
| CD200 | NM_001004196 | | CD200 molecule | **-0.98** | 0.019 |
| KIR2DS4 | NM_012314 | | killer cell immunoglobulin-like receptor, two domains, short cytoplasmic tail, 4 | **-0.98** | 0.012 |
| PSD3 | NM_015310 | | pleckstrin and Sec7 domain containing 3 | **-0.98** | 0.042 |
| GK | NM_203391 | | glycerol kinase | **-0.98** | 0.011 |
| FOLR4 | NM_001080486 | | similar to folate receptor 4 (delta) isoform 1 | **-0.98** | 0.020 |
| PACSIN1 | NM_020804 | | protein kinase C and casein kinase substrate in neurons 1 | **-0.97** | 0.031 |
| ABCA10 | NM_080282 | | ATP-binding cassette, sub-family A (ABC1), member 10 | **-0.97** | 0.032 |
|  | M15530 | | B-cell growth factor (BCGF1) | **-0.96** | 0.006 |
| EVPLL | NM_001145127 | | envoplakin-like | **-0.96** | 0.008 |
| VEGFA | NM_001025366 | | vascular endothelial growth factor A | **-0.96** | 0.017 |
|  | AK000707 | | Unknown | **-0.96** | 0.028 |
| KGFLP1 | AY098593 | | keratinocyte growth factor-like protein 1 | **-0.95** | 0.010 |
|  | BC031342 | | CDNA FLJ12777 fis, clone NT2RP2001720 | **-0.95** | 0.019 |
| C11orf35 | NM_173573 | | chromosome 11 open reading frame 35 | **-0.94** | 0.018 |
| KY | NM_178554 | | kyphoscoliosis peptidase | **-0.94** | 0.039 |
| HTR3A | NM_213621 | | 5-hydroxytryptamine (serotonin) receptor 3A | **-0.94** | 0.021 |
|  | THC2727342 | | Unknown | **-0.93** | 0.029 |
|  | AL833005 | | Transcribed locus | **-0.93** | 0.025 |
|  | S79672 | | Unknown | **-0.92** | 0.036 |
| IL7 | NM_000880 | | interleukin 7 | **-0.92** | 0.023 |
| HK3 | NM_002115 | | hexokinase 3 (white cell) | **-0.92** | 0.006 |
| NDRG1 | NM_006096 | | N-myc downstream regulated gene 1 | **-0.92** | 0.014 |
| PRKACG | NM_002732 | | protein kinase, cAMP-dependent, catalytic, gamma | **-0.92** | 0.024 |
| SPACA1 | NM_030960 | | sperm acrosome associated 1 | **-0.91** | 0.018 |
|  | AK022168 | | CDNA FLJ12106 fis, clone HEMBB1002702 | **-0.91** | 0.049 |
| C3orf47 | NR_026991 | | chromosome 3 open reading frame 47 | **-0.91** | 0.027 |
|  | AK125672 | | Unknown | **-0.91** | 0.023 |
|  | AL833005 | | Transcribed locus | **-0.91** | 0.024 |
| PP8961 | NM_001080113 | | hypothetical protein LOC650662 | **-0.91** | 0.022 |
| PRCD | NM_001077620 | | progressive rod-cone degeneration | **-0.91** | 0.020 |
|  | XR_018565 | | isocitrate dehydrogenase pseudogene | **-0.91** | 0.032 |
| MRC1L1 | NM_001009567 | | mannose receptor, C type 1-like 1 | **-0.90** | 0.019 |
|  | XR_019164 | | similar to RalA-binding protein 1 (RalBP1) | **-0.90** | 0.046 |
| SMOX | NM_175839 | | spermine oxidase | **-0.90** | 0.007 |
| SNX26 | AL566332 | | Sorting nexin 26 | **-0.90** | 0.011 |
| FAM65C | NM_080829 | | chromosome 20 open reading frame 175 | **-0.90** | 0.021 |
|  | BC032409 | | Transcribed locus, weakly similar to XP_537423.2 PREDICTED: similar to LINE-1 reverse transcriptase homolog [Canis familiaris] | **-0.89** | 0.025 |
| OR14A16 | NM_001001966 | | olfactory receptor, family 14, subfamily A, member 16 | **-0.88** | 0.018 |
| CSHL1 | NM_022579 | | chorionic somatomammotropin hormone-like 1 | **-0.88** | 0.018 |
| LOC493754 | AK090474 | | hypothetical LOC441245 | **-0.88** | 0.022 |
|  | AB062480 | | OK/SW-cl.79 | **-0.88** | 0.032 |
| BDKRB2 | NM_000623 | | bradykinin receptor B2 | **-0.88** | 0.006 |
| SEC31A | AK128047 | | SEC31 homolog A (S. cerevisiae) | **-0.88** | 0.018 |
|  | ENST00000342294 | | Unknown | **-0.87** | 0.017 |
| FANCC | BC034271 | | Fanconi anemia, complementation group C | **-0.87** | 0.034 |
|  | XR_019354 | | similar to DnaJ (Hsp40) homolog, subfamily C, member 7 | **-0.87** | 0.036 |
| NUBPL | NM_025152 | | nucleotide binding protein-like | **-0.87** | 0.034 |
|  | BC032042 | | CDNA clone IMAGE:4819488 | **-0.87** | 0.040 |
| SLC38A2 | NM_018976 | | solute carrier family 38, member 2 | **-0.87** | 0.007 |
| LOC401357 | NM_001013685 | | hypothetical LOC401357 | **-0.87** | 0.028 |
| GSTTP2 | NR_003082 | | glutathione S-transferase theta pseudogene 2 | **-0.87** | 0.021 |
| COL12A1 | NM_004370 | | collagen, type XII, alpha 1 | **-0.87** | 0.016 |
| C17orf60 | ENST00000332935 | | chromosome 17 open reading frame 60 | **-0.86** | 0.024 |
| C15orf28 | AK021784 | | chromosome 15 open reading frame 28 | **-0.86** | 0.001 |
| OR4F5 | NM_001005484 | | olfactory receptor, family 4, subfamily F, member 5 | **-0.86** | 0.024 |
|  | AL049991 | | MRNA; cDNA DKFZp564G222 | **-0.85** | 0.038 |
|  | BC033590 | | Homo sapiens, clone IMAGE:4344825, mRNA | **-0.85** | 0.013 |
| EFCAB4A | NM_173584 | | EF-hand calcium binding domain 4A | **-0.85** | 0.007 |
|  | AI240896 | | Transcribed locus | **-0.85** | 0.022 |
|  | AL512741 | | MRNA; cDNA DKFZp667N064 | **-0.85** | 0.021 |
| RBP5 | NM_031491 | | retinol binding protein 5, cellular | **-0.84** | 0.024 |
| BSX | NM_001098169 | | brain-specific homeobox | **-0.84** | 0.012 |
| HIST1H2AE | NM_021052 | | histone cluster 1, H2ae | **-0.83** | 0.039 |
|  | THC2485189 | | Unknown | **-0.83** | 0.019 |
|  | NM_181722 | | hypothetical protein LOC285908 | **-0.83** | 0.047 |
| ETV1 | NM_004956 | | ets variant gene 1 | **-0.83** | 0.030 |
|  | NR_003319 | | small nucleolar RNA, C/D box 116-4 | **-0.83** | 0.047 |
| LIMS3 | NM_033514 | | LIM and senescent cell antigen-like domains 3 | **-0.83** | 0.041 |
| LAMA4 | NM_002290 | | laminin, alpha 4 | **-0.82** | 0.000 |
| DUSP2 | NM_004418 | | dual specificity phosphatase 2 | **-0.82** | 0.020 |
|  | THC2701763 | | Unknown | **-0.82** | 0.041 |
|  | THC2644672 | | Unknown | **-0.81** | 0.040 |
| C2orf67 | NM_152519 | | hypothetical protein FLJ23861 | **-0.81** | 0.023 |
| DDI2 | BC006011 | | DDI1, DNA-damage inducible 1, homolog 2 (S. cerevisiae) | **-0.81** | 0.023 |
|  | BE467780 | | Transcribed locus | **-0.80** | 0.025 |
| SPRED3 | NM_001039616 | | sprouty-related, EVH1 domain containing 3 | **-0.80** | 0.002 |
| C22orf34 | NR_026997 | | chromosome 22 open reading frame 34 | **-0.80** | 0.050 |
| XAGE3 | NM_130776 | | X antigen family, member 3 | **-0.80** | 0.041 |
|  | BP871540 | | Transcribed locus | **-0.80** | 0.021 |
| FRMD4A | AK001072 | | FERM domain containing 4A | **-0.80** | 0.003 |
| REG3A | NM_138938 | | regenerating islet-derived 3 alpha | **-0.79** | 0.038 |
| LOC100131582 | CR594528 | | Hypothetical protein LOC100131582 | **-0.79** | 0.010 |
| WWTR1 | AL050107 | | WW domain containing transcription regulator 1 | **-0.78** | 0.037 |
|  | AL136837 | | hypothetical protein DKFZp434F142 | **-0.78** | 0.010 |
|  | XM_927380 | | hypothetical protein LOC644186 | **-0.78** | 0.041 |
| GRIA3 | NM_000828 | | glutamate receptor, ionotrophic, AMPA 3 | **-0.78** | 0.034 |
| UTF1 | NM_003577 | | undifferentiated embryonic cell transcription factor 1 | **-0.78** | 0.019 |
| OCM2 | NM_006188 | | Oncomodulin | **-0.78** | 0.042 |
| ZFP92 | NM_001136273 | | zinc finger protein 92 homolog (mouse) | **-0.77** | 0.043 |
| RP13-388O5.1 | NM_001080483 | | transmembrane protein 8C | **-0.77** | 0.032 |
| P2RX1 | NM_002558 | | purinergic receptor P2X, ligand-gated ion channel, 1 | **-0.77** | 0.026 |
|  | XR_018063 | | hypothetical LOC392030 | **-0.77** | 0.004 |
|  | NR_015389 | | hypothetical LOC339290 | **-0.77** | 0.033 |
|  | THC2523793 | | Unknown | **-0.77** | 0.034 |
|  | THC2558699 | | Unknown | **-0.76** | 0.021 |
| C2orf14 | AL136789 | | chromosome 2 open reading frame 14 | **-0.76** | 0.022 |
|  | NR_003321 | | small nucleolar RNA, C/D box 116-6 | **-0.76** | 0.035 |
| CCDC108 | NM_194302 | | coiled-coil domain containing 108 | **-0.76** | 0.024 |
|  | NR_003187 | | neutrophil cytosolic factor 1C pseudogene | **-0.76** | 0.036 |
| LCE2C | NM_178429 | | late cornified envelope 2C | **-0.75** | 0.025 |
| PLA2G4A | NM_024420 | | phospholipase A2, group IVA (cytosolic, calcium-dependent) | **-0.75** | 0.022 |
| APOC1 | NM_001645 | | apolipoprotein C-I | **-0.74** | 0.046 |
| LOC100131551 | NR_024480 | | hypothetical LOC100131551 | **-0.74** | 0.035 |
| PTMS | NM_002824 | | Parathymosin | **-0.74** | 0.007 |
| LOC100132244 | AK026140 | | Hypothetical protein LOC100132244 | **-0.74** | 0.039 |
| STAR | NM_000349 | | steroidogenic acute regulatory protein | **-0.74** | 0.027 |
| ZFX | NM_003410 | | zinc finger protein, X-linked | **-0.73** | 0.039 |
| LOC646603 | NM_001080531 | | hypothetical LOC646603 | **-0.73** | 0.037 |
| RPL21 | BC104478 | | ribosomal protein L21 | **-0.73** | 0.010 |
| VSX1 | NM_014588 | | visual system homeobox 1 homolog, CHX10-like (zebrafish) | **-0.73** | 0.029 |
|  | BC031954 | | CDNA clone IMAGE:4828714 | **-0.73** | 0.033 |
| C8A | NM_000562 | | complement component 8, alpha polypeptide | **-0.72** | 0.048 |
| TSC22D1 | NM_183422 | | TSC22 domain family, member 1 | **-0.72** | 0.028 |
|  | AW015426 | | Transcribed locus | **-0.72** | 0.045 |
| ODF4 | NM_153007 | | outer dense fiber of sperm tails 4 | **-0.72** | 0.042 |
| PPFIBP1 | NM_003622 | | PTPRF interacting protein, binding protein 1 (liprin beta 1) | **-0.71** | 0.011 |
|  | ENST00000375284 | | Unknown | **-0.70** | 0.043 |
|  | AL834342 | | Transcribed locus | **-0.70** | 0.036 |
| SLC16A4 | NM_004696 | | solute carrier family 16, member 4 (monocarboxylic acid transporter 5) | **-0.70** | 0.032 |
|  | AK097080 | | CDNA FLJ52612 complete cds | **-0.70** | 0.042 |
|  | AA814210 | | Transcribed locus | **-0.70** | 0.047 |
| TRPV6 | NM_018646 | | transient receptor potential cation channel, subfamily V, member 6 | **-0.69** | 0.039 |
| LOC338620 | BC043009 | | hypothetical protein LOC338620 | **-0.69** | 0.018 |
|  | U25029 | | Glucocorticoid receptor alpha mRNA, variant 3' UTR | **-0.69** | 0.022 |
|  | AW817085 | | Transcribed locus | **-0.68** | 0.031 |
| MS4A5 | NM_023945 | | membrane-spanning 4-domains, subfamily A, member 5 | **-0.68** | 0.042 |
| FXYD7 | NM_022006 | | FXYD domain containing ion transport regulator 7 | **-0.68** | 0.047 |
| FAM78A | NM_033387 | | family with sequence similarity 78, member A | **-0.68** | 0.049 |
|  | AA975908 | | Transcribed locus | **-0.68** | 0.040 |
| ZFATAS | NR_002438 | | ZFAT antisense RNA (non-protein coding) | **-0.67** | 0.038 |
| FOXP1 | NM_032682 | | forkhead box P1 | **-0.67** | 0.026 |
| IRF2BP2 | NM_182972 | | interferon regulatory factor 2 binding protein 2 | **-0.67** | 0.034 |
| LOC150759 | AK057596 | | hypothetical protein LOC150759 | **-0.67** | 0.037 |
| OTOF | NM_194248 | | Otoferlin | **-0.67** | 0.039 |
| CCDC74B | NM_207310 | | coiled-coil domain containing 74B | **-0.67** | 0.036 |
| DHRS3 | NM_004753 | | dehydrogenase/reductase (SDR family) member 3 | **-0.67** | 0.032 |
| FAM123C | NM_152698 | | hypothetical protein FLJ38377 | **-0.67** | 0.046 |
| CSF3R | NM_172313 | | colony stimulating factor 3 receptor (granulocyte) | **-0.66** | 0.043 |
| DUSP4 | NM_001394 | | dual specificity phosphatase 4 | **-0.65** | 0.048 |
| TMEM129 | NM_138385 | | transmembrane protein 129 | **-0.65** | 0.027 |
| NCRNA00112 | NR_024028 | | non-protein coding RNA 112 | **-0.65** | 0.049 |
| PIK3R5 | NM_014308 | | phosphoinositide-3-kinase, regulatory subunit 5, p101 | **-0.65** | 0.048 |
| HNF1A | NM_000545 | | hepatic nuclear factor (HNF1) | **-0.64** | 0.035 |
| ROBO1 | NM_133631 | | roundabout, axon guidance receptor, homolog 1 (Drosophila) | **-0.63** | 0.043 |
|  | AF086288 | | Full length insert cDNA clone ZD48A05 | **-0.63** | 0.049 |
| GABRE | NM_021990 | | gamma-aminobutyric acid (GABA) A receptor, epsilon | **-0.63** | 0.016 |
|  | ENST00000328411 | | Unknown | **-0.62** | 0.046 |
| FSHR | NM_000145 | | follicle stimulating hormone receptor | **-0.62** | 0.045 |
| PTPN18 | NM_014369 | | protein tyrosine phosphatase, non-receptor type 18 (brain-derived) | **-0.61** | 0.025 |
|  | AK026367 | | Unknown | **-0.61** | 0.042 |
| KIAA1875 | NM_032529 | | KIAA1875 | **-0.60** | 0.049 |
