# Supplementary material for: High Throughput Transcriptome Profiling of Lithium Stimulated Human Mesenchymal Stem Cells Reveals Priming towards Osteoblastic Lineage
Source: PLoS One. 2013 Jan 30;8(1):e55769. doi: 10.1371/journal.pone.0055769 (PMC3559497; doi:10.1371/journal.pone.0055769)
Supplement: Table S4 — Biological Processes Classification of Significantly Regulated Genes. (DOC) [file pone.0055769.s005.doc]

Table S4: Biological Processes Classification of Significantly Regulated Genes

| **GO ID** | **Term** | **No. of genes in our list** | **Total no. of genes** | **P Value** |
| --- | --- | --- | --- | --- |
| **UPREGULATED GENES** | | | | |
| GO:0007565 | female pregnancy | 6 | 110 | 4.63E-05 |
| GO:0007165 | signal transduction | 22 | 2840 | 4.70E-04 |
| GO:0014033 | neural crest cell differentiation | 3 | 33 | 0.006291 |
| GO:0014032 | neural crest cell development | 3 | 33 | 0.006291 |
| GO:0043179 | rhythmic excitation | 2 | 2 | 0.007213 |
| GO:0007606 | sensory perception of chemical stimulus | 7 | 478 | 0.00724 |
| GO:0050896 | response to stimulus | 22 | 3502 | 0.007361 |
| GO:0051704 | multi-organism process | 8 | 681 | 0.010757 |
| GO:0060024 | rhythmic synaptic transmission | 2 | 3 | 0.0108 |
| GO:0014031 | mesenchymal cell development | 3 | 51 | 0.014576 |
| GO:0048762 | mesenchymal cell differentiation | 3 | 51 | 0.014576 |
| GO:0060485 | mesenchyme development | 3 | 52 | 0.015124 |
| GO:0050890 | cognition | 9 | 909 | 0.015479 |
| GO:0007186 | G-protein coupled receptor protein signaling pathway | 10 | 1123 | 0.018021 |
| GO:0022414 | reproductive process | 8 | 762 | 0.018974 |
| GO:0007608 | sensory perception of smell | 6 | 431 | 0.019251 |
| GO:0000003 | reproduction | 8 | 767 | 0.019596 |
| GO:0008283 | cell proliferation | 6 | 436 | 0.020128 |
| GO:0034637 | cellular carbohydrate biosynthetic process | 3 | 69 | 0.025739 |
| GO:0050877 | neurological system process | 10 | 1210 | 0.027794 |
| GO:0007166 | cell surface receptor linked signal transduction | 13 | 1856 | 0.030031 |
| GO:0051046 | regulation of secretion | 4 | 202 | 0.036398 |
| GO:0008361 | regulation of cell size | 4 | 206 | 0.038232 |
| GO:0003008 | system process | 11 | 1503 | 0.040247 |
| GO:0050794 | regulation of cellular process | 32 | 6819 | 0.04969 |
| **DOWNREGULATED GENES** | | | | |
| GO:0001894 | tissue homeostasis | 7 | 63 | 2.85E-05 |
| GO:0009605 | response to external stimulus | 23 | 914 | 4.72E-05 |
| GO:0048875 | chemical homeostasis within a tissue | 4 | 10 | 9.56E-05 |
| GO:0043129 | surfactant homeostasis | 4 | 10 | 9.56E-05 |
| GO:0032501 | multicellular organismal process | 62 | 4280 | 1.40E-04 |
| GO:0048871 | multicellular organismal homeostasis | 7 | 85 | 1.55E-04 |
| GO:0048608 | reproductive structure development | 8 | 126 | 1.96E-04 |
| GO:0003006 | reproductive developmental process | 11 | 262 | 1.98E-04 |
| GO:0006576 | biogenic amine metabolic process | 7 | 97 | 3.19E-04 |
| GO:0048731 | system development | 39 | 2330 | 3.83E-04 |
| GO:0042221 | response to chemical stimulus | 26 | 1281 | 3.87E-04 |
| GO:0007275 | multicellular organismal development | 45 | 2865 | 4.28E-04 |
| GO:0060249 | anatomical structure homeostasis | 7 | 106 | 5.13E-04 |
| GO:0008406 | gonad development | 7 | 112 | 6.87E-04 |
| GO:0050927 | positive regulation of positive chemotaxis | 4 | 19 | 7.25E-04 |
| GO:0050926 | regulation of positive chemotaxis | 4 | 19 | 7.25E-04 |
| GO:0032502 | developmental process | 47 | 3148 | 9.24E-04 |
| GO:0008610 | lipid biosynthetic process | 11 | 323 | 0.00102 |
| GO:0006575 | cellular amino acid derivative metabolic process | 8 | 166 | 0.001031 |
| GO:0045137 | development of primary sexual characteristics | 7 | 127 | 0.001323 |
| GO:0048511 | rhythmic process | 7 | 128 | 0.001377 |
| GO:0044255 | cellular lipid metabolic process | 14 | 526 | 0.001486 |
| GO:0048513 | organ development | 30 | 1738 | 0.001602 |
| GO:0048856 | anatomical structure development | 39 | 2527 | 0.00185 |
| GO:0060627 | regulation of vesicle-mediated transport | 6 | 96 | 0.002192 |
| GO:0050921 | positive regulation of chemotaxis | 4 | 29 | 0.002551 |
| GO:0022602 | ovulation cycle process | 5 | 62 | 0.00283 |
| GO:0050920 | regulation of chemotaxis | 4 | 31 | 0.003096 |
| GO:0007548 | sex differentiation | 7 | 151 | 0.003169 |
| GO:0032103 | positive regulation of response to external stimulus | 5 | 64 | 0.003176 |
| GO:0008585 | female gonad development | 5 | 64 | 0.003176 |
| GO:0010035 | response to inorganic substance | 8 | 205 | 0.003415 |
| GO:0042698 | ovulation cycle | 5 | 67 | 0.003749 |
| GO:0050930 | induction of positive chemotaxis | 3 | 10 | 0.003829 |
| GO:0048520 | positive regulation of behavior | 4 | 34 | 0.004037 |
| GO:0046660 | female sex differentiation | 5 | 69 | 0.004167 |
| GO:0046545 | development of primary female sexual characteristics | 5 | 69 | 0.004167 |
| GO:0042330 | taxis | 7 | 160 | 0.00421 |
| GO:0006935 | chemotaxis | 7 | 160 | 0.00421 |
| GO:0007626 | locomotory behavior | 9 | 274 | 0.004621 |
| GO:0050896 | response to stimulus | 48 | 3502 | 0.004916 |
| GO:0009991 | response to extracellular stimulus | 8 | 220 | 0.005018 |
| GO:0006955 | immune response | 15 | 690 | 0.00589 |
| GO:0048609 | reproductive process in a multicellular organism | 12 | 487 | 0.006683 |
| GO:0032504 | multicellular organism reproduction | 12 | 487 | 0.006683 |
| GO:0001541 | ovarian follicle development | 4 | 41 | 0.006853 |
| GO:0050679 | positive regulation of epithelial cell proliferation | 4 | 41 | 0.006853 |
| GO:0045017 | glycerolipid biosynthetic process | 5 | 80 | 0.007039 |
| GO:0002684 | positive regulation of immune system process | 8 | 238 | 0.007625 |
| GO:0010038 | response to metal ion | 6 | 129 | 0.007706 |
| GO:0002687 | positive regulation of leukocyte migration | 3 | 15 | 0.008662 |
| GO:0050795 | regulation of behavior | 4 | 46 | 0.009429 |
| GO:0006690 | icosanoid metabolic process | 4 | 47 | 0.010003 |
| GO:0006629 | lipid metabolic process | 16 | 813 | 0.010223 |
| GO:0007584 | response to nutrient | 6 | 140 | 0.010754 |
| GO:0031667 | response to nutrient levels | 7 | 197 | 0.01126 |
| GO:0009611 | response to wounding | 12 | 530 | 0.012146 |
| GO:0033559 | unsaturated fatty acid metabolic process | 4 | 51 | 0.0125 |
| GO:0032879 | regulation of localization | 13 | 610 | 0.013266 |
| GO:0022414 | reproductive process | 15 | 762 | 0.01342 |
| GO:0001889 | liver development | 4 | 53 | 0.01387 |
| GO:0040017 | positive regulation of locomotion | 5 | 98 | 0.014117 |
| GO:0000003 | reproduction | 15 | 767 | 0.01414 |
| GO:0030324 | lung development | 5 | 99 | 0.014606 |
| GO:0048518 | positive regulation of biological process | 30 | 2033 | 0.014732 |
| GO:0002685 | regulation of leukocyte migration | 3 | 20 | 0.015196 |
| GO:0048771 | tissue remodeling | 4 | 56 | 0.016079 |
| GO:0030323 | respiratory tube development | 5 | 102 | 0.016136 |
| GO:0008654 | phospholipid biosynthetic process | 5 | 102 | 0.016136 |
| GO:0008284 | positive regulation of cell proliferation | 10 | 414 | 0.0172 |
| GO:0032101 | regulation of response to external stimulus | 6 | 159 | 0.017796 |
| GO:0035295 | tube development | 7 | 220 | 0.01849 |
| GO:0007154 | cell communication | 15 | 795 | 0.018735 |
| GO:0046486 | glycerolipid metabolic process | 6 | 162 | 0.019132 |
| GO:0060541 | respiratory system development | 5 | 108 | 0.01949 |
| GO:0001775 | cell activation | 8 | 287 | 0.019669 |
| GO:0006979 | response to oxidative stress | 6 | 164 | 0.020059 |
| GO:0040011 | locomotion | 10 | 431 | 0.021697 |
| GO:0010243 | response to organic nitrogen | 4 | 63 | 0.021962 |
| GO:0065007 | biological regulation | 84 | 7484 | 0.022681 |
| GO:0009888 | tissue development | 13 | 665 | 0.024323 |
| GO:0033273 | response to vitamin | 4 | 66 | 0.024796 |
| GO:0048584 | positive regulation of response to stimulus | 7 | 236 | 0.02508 |
| GO:0045944 | positive regulation of transcription from RNA polymerase II promoter | 9 | 371 | 0.025161 |
| GO:0046474 | glycerophospholipid biosynthetic process | 4 | 68 | 0.02679 |
| GO:0002376 | immune system process | 17 | 998 | 0.027055 |
| GO:0007267 | cell-cell signaling | 12 | 600 | 0.0276 |
| GO:0045321 | leukocyte activation | 7 | 242 | 0.02791 |
| GO:0031328 | positive regulation of cellular biosynthetic process | 13 | 685 | 0.029685 |
| GO:0050678 | regulation of epithelial cell proliferation | 4 | 71 | 0.029937 |
| GO:0008544 | epidermis development | 6 | 184 | 0.030965 |
| GO:0051239 | regulation of multicellular organismal process | 16 | 937 | 0.032269 |
| GO:0009891 | positive regulation of biosynthetic process | 13 | 695 | 0.032668 |
| GO:0048583 | regulation of response to stimulus | 10 | 465 | 0.033132 |
| GO:0007610 | behavior | 10 | 469 | 0.034709 |
| GO:0048661 | positive regulation of smooth muscle cell proliferation | 3 | 31 | 0.034759 |
| GO:0042060 | wound healing | 6 | 191 | 0.035516 |
| GO:0040012 | regulation of locomotion | 6 | 192 | 0.036198 |
| GO:0031325 | positive regulation of cellular metabolic process | 15 | 880 | 0.039744 |
| GO:0042439 | ethanolamine and derivative metabolic process | 3 | 34 | 0.041173 |
| GO:0007398 | ectoderm development | 6 | 199 | 0.0412 |
| GO:0048522 | positive regulation of cellular process | 26 | 1847 | 0.041236 |
| GO:0019637 | organophosphate metabolic process | 6 | 200 | 0.041947 |
| GO:0006950 | response to stress | 24 | 1685 | 0.045894 |
| GO:0010557 | positive regulation of macromolecule biosynthetic process | 12 | 654 | 0.046811 |
