# Supplementary material for: High Throughput Transcriptome Profiling of Lithium Stimulated Human Mesenchymal Stem Cells Reveals Priming towards Osteoblastic Lineage
Source: PLoS One. 2013 Jan 30;8(1):e55769. doi: 10.1371/journal.pone.0055769 (PMC3559497; doi:10.1371/journal.pone.0055769)
Supplement: Table S5 — Signaling And Metabolic Pathways Represented By Differentially Regulated Genes. (DOC) [file pone.0055769.s006.doc]

**Table S5: Signaling And Metabolic Pathways Represented By Differentially Regulated Genes**

| **Pathway Name** | **Number of Genes Found in the Pathway** | **Genes in the Pathway** | **p value** |
| --- | --- | --- | --- |
| **UPREGULATED GENES** | | | |
| Hs GPCRDB Class A Rhodopsin-like (GenMAPP) | 3 | OXTR, OR10H1, ARHGDIB | 0.00074 |
| MAPK signaling pathway (KEGG) | 4 | FLNC, ATF4, RRAS2, ELK4 | 0.00096 |
| Tight junction (KEGG) | 3 | RRAS2, MPP5, MLLT4 | 0.00598 |
| Neuroactive ligand-receptor interaction (KEGG) | 3 | GRPR, OXTR, EDN1 | 0.00598 |
| Hs Glycolysis and Gluconeogenesis (GenMAPP) | 2 | RRAS2, ALDOB | 0.00932 |
| Hs Smooth muscle contraction (GenMAPP) | 2 | ATF4, OXTR | 0.00932 |
| Long-term potentiation (KEGG) | 2 | ATF4, RRAS2 | 0.03497 |
| Focal adhesion (KEGG) | 2 | FLNC, RRAS2 | 0.03497 |
| Hematopoietic cell lineage (KEGG) | 2 | IL7R, CD36 | 0.03497 |
| Cytokine-cytokine receptor interaction (KEGG) | 2 | IL7R, CD70 | 0.03497 |
| Calcium signaling pathway (KEGG) | 2 | GRPR, OXTR | 0.03497 |
| **DOWNREGULATED GENES** | | | |
| MAPK signaling pathway (KEGG) | 7 | NR4A1, FGF7, CACNG6, PLA2G4A, PRKACG, DUSP4, DUSP2 | 0.00004 |
| Cytokine-cytokine receptor interaction (KEGG) | 7 | CXCL12, HGF, CCL20, CXCL1, CSF3R, VEGFA, IL7 | 0.00004 |
| Neuroactive ligand-receptor interaction (KEGG) | 6 | HTR7, P2RX1, BDKRB2, GABRE, GRIA3, FSHR | 0.00017 |
| Hs GPCRDB Class A Rhodopsin-like (GenMAPP) | 3 | OR2W1, HTR7, FSHR | 0.00062 |
| Hs Prostaglandin synthesis regulation (GenMAPP) | 3 | PTGS2, HSD11B1, PLA2G4A | 0.00062 |
| Focal adhesion (KEGG) | 5 | ITGA2, HGF, LAMA4, VEGFA, PIK3R5 | 0.00076 |
| Hs Irinotecan pathway PharmGKB (GenMAPP) | 2 | PTGS2, PTGES | 0.00765 |
| Hs Glycolysis and Gluconeogenesis (GenMAPP) | 2 | HK3, PIK3R5 | 0.00765 |
| Hs GPCRDB Other (GenMAPP) | 2 | HTR7, FSHR | 0.00765 |
| Hs Nuclear Receptors (GenMAPP) | 2 | NR4A1, RXRG | 0.00765 |
| Actions of Nitric Oxide in the Heart (Biocarta) | 3 | BDKRB2, PRKACG, VEGFA | 0.00147 |
| Eicosanoid Metabolism (Biocarta) | 3 | PTGS2, PTGES, PLA2G4A | 0.00147 |
| Arachidonic acid metabolism (KEGG) | 4 | PTGS2, PTGES, CYP4F2, PLA2G4A | 0.00331 |
| Complement and coagulation cascades (KEGG) | 4 | THBD, CFI, BDKRB2, C8A | 0.00331 |
| Regulation of actin cytoskeleton (KEGG) | 4 | ITGA2, FGF7, BDKRB2, PIK3R5 | 0.00331 |
| Leukocyte transendothelial migration (KEGG) | 4 | CXCL12, PECAM1, PIK3R5, CLDN20 | 0.00331 |
| Calcium signaling pathway (KEGG) | 4 | HTR7, P2RX1, BDKRB2, PRKACG | 0.00331 |
| Neuropeptides VIP and PACAP inhibit the apoptosis of activated T cells (Biocarta) | 2 | EGR3, PRKACG | 0.01341 |
| Mechanism of Gene Regulation by Peroxisome Proliferators via PPARa(alpha) (Biocarta) | 2 | PTGS2, PRKACG | 0.01341 |
| Regulation of MAP Kinase Pathways Through Dual Specificity Phosphatases (Biocarta) | 2 | DUSP4, DUSP2 | 0.01341 |
| Cell adhesion molecules (CAMs) (KEGG) | 3 | PECAM1, CD86, CLDN20 | 0.01412 |
| VEGF signaling pathway (KEGG) | 3 | PLA2G4A, VEGFA, PIK3R5 | 0.01412 |
| Hematopoietic cell lineage (KEGG) | 3 | ITGA2, CSF3R, IL7 | 0.01412 |
| Jak-STAT signaling pathway (KEGG) | 3 | CSF3R, PIK3R5, IL7 | 0.01412 |
